# Evaluation of the effectiveness of topical repellent distributed by village health volunteer networks against *Plasmodium* spp. infection in Myanmar: A stepped-wedge cluster randomised trial

**DOI:** 10.1371/journal.pmed.1003177

**Published:** 2020-08-20

**Authors:** Paul A. Agius, Julia C. Cutts, Win Han Oo, Aung Thi, Katherine O’Flaherty, Kyaw Zayar Aung, Htin Kyaw Thu, Poe Poe Aung, Myat Mon Thein, Nyi Nyi Zaw, Wai Yan Min Htay, Aung Paing Soe, Zahra Razook, Alyssa E. Barry, Win Htike, Angela Devine, Julie A. Simpson, Brendan S. Crabb, James G. Beeson, Naanki Pasricha, Freya J. I. Fowkes

**Affiliations:** 1 Burnet Institute, Victoria, Australia, and Yangon, Myanmar; 2 Department of Epidemiology and Preventive Medicine, Monash University, Melbourne, Victoria, Australia; 3 Department of Public Health, Myanmar Ministry of Health and Sports, Nay Pyi Taw, Myanmar; 4 Centre for Epidemiology and Biostatistics, Melbourne School of Population and Global Health, University of Melbourne, Melbourne, Victoria, Australia; 5 Population Health and Immunity Division, Walter and Eliza Hall Institute of Medical Research, Melbourne, Victoria, Australia; 6 Global Health Division, Department of Medical Biology, University of Melbourne, Melbourne, Victoria, Australia; 7 Menzies School of Health Research, Charles Darwin University, Darwin, Northern Territory, Australia; 8 Department of Medicine, University of Melbourne, Melbourne, Victoria, Australia; 9 Central Clinical School, Monash University, Melbourne, Victoria, Australia; Mahidol-Oxford Tropical Medicine Research Unit, THAILAND

## Abstract

**Background:**

The World Health Organization has yet to endorse deployment of topical repellents for malaria prevention as part of public health campaigns. We aimed to quantify the effectiveness of repellent distributed by the village health volunteer (VHV) network in the Greater Mekong Subregion (GMS) in reducing malaria in order to advance regional malaria elimination.

**Methods and findings:**

Between April 2015 and June 2016, a 15-month stepped-wedge cluster randomised trial was conducted in 116 villages in Myanmar (stepped monthly in blocks) to test the effectiveness of 12% N,N-diethylbenzamide w/w cream distributed by VHVs, on *Plasmodium* spp. infection. The median age of participants was 18 years, approximately half were female, and the majority were either village residents (46%) or forest dwellers (40%). No adverse events were reported during the study. Generalised linear mixed modelling estimated the effect of repellent on infection detected by rapid diagnostic test (RDT) (primary outcome) and polymerase chain reaction (PCR) (secondary outcome). Overall *Plasmodium* infection detected by RDT was low (0.16%; 50/32,194), but infection detected by PCR was higher (3%; 419/13,157). There was no significant protection against RDT-detectable infection (adjusted odds ratio [AOR] = 0.25, 95% CI 0.004–15.2, *p =* 0.512). In *Plasmodium-*species-specific analyses, repellent protected against PCR-detectable *P*. *falciparum* (adjusted relative risk ratio [ARRR] = 0.67, 95% CI 0.47–0.95, *p =* 0.026), but not *P*. *vivax* infection (ARRR = 1.41, 95% CI 0.80–2.47, *p =* 0.233). Repellent effects were similar when delayed effects were modelled, across risk groups, and regardless of village-level and temporal heterogeneity in malaria prevalence. The incremental cost-effectiveness ratio was US$256 per PCR-detectable infection averted. Study limitations were a lower than expected *Plasmodium* spp. infection rate and potential geographic dilution of the intervention.

**Conclusions:**

In this study, we observed apparent protection against new infections associated with the large-scale distribution of repellent by VHVs. Incorporation of repellent into national strategies, particularly in areas where bed nets are less effective, may contribute to the interruption of malaria transmission. Further studies are warranted across different transmission settings and populations, from the GMS and beyond, to inform WHO public health policy on the deployment of topical repellents for malaria prevention.

**Trial registration:**

Australian and New Zealand Clinical Trials Registry (ACTRN12616001434482).

## Abstract 2

Paul Agius and colleagues study a topical insect repellent for protection against malaria.

## Author summary

**Why was this study done?:**

- Despite there being strong evidence for efficacy of topical repellent in preventing mosquito bites, there is a paucity of high-quality evidence for the effectiveness of repellent in reducing malaria.
- Topical repellents may be highly relevant in the Greater Mekong Subregion (GMS), where malaria vectors bite outdoors and are not confined to biting at night (and thus not covered by conventional vector control measures).
- We wanted to know whether distributing repellent through established village health volunteer (VHV) networks in the GMS would prevent malaria infection.

**What did the researchers do and find?:**

- We randomised 116 villages in Myanmar to receive repellent through VHV networks using a stepped-wedge design and followed the villages up for 15 months to detect malaria by rapid diagnostic test (RDT) and highly sensitive polymerase chain reaction (PCR).
- We found that distribution of repellent through the VHV network did not reduce RDT-detectable infection, but appeared to reduce the odds of PCR-detectable infection.
- The apparent protective effect of repellent was consistent across risk groups (village residents, forest dwellers, and migrants) and villages, suggesting that repellent distributed by VHVs may be an effective intervention across a range of transmission settings and populations.

**What do these findings mean?:**

- Distribution of topical repellent by the VHV network may be an effective supplementary intervention for reducing malaria in the GMS.
- Incorporation of repellent into national malaria control programme national strategic plans for malaria elimination may advance achieving GMS malaria elimination targets of 2025 for *P*. *falciparum* and 2030 for all malaria species, particularly in the context of limited effectiveness of traditional vector control interventions and spreading drug resistance.
- Further studies are warranted across different transmission settings and populations, from the GMS and beyond, to inform World Health Organization public health policy on the deployment of topical repellents for malaria prevention.

## Introduction

While there have been major gains in reducing the global burden of malaria since the turn of the millennium, the progress in reducing malaria has recently stalled [1]. Vector control interventions, namely insecticide-treated bed nets (ITNs) and indoor residual spraying (IRS) have contributed significantly to the reductions in malaria globally [1]. However, increasing rates of insecticide resistance and changes in vector composition and behaviour (changing biting hours and preference for outdoor biting) have reduced the effectiveness of these cornerstone vector control interventions [2,3]. Evidence for additional tools that target residual transmission not covered by ITNs and IRS is needed in order to achieve malaria control and elimination goals.

Although there is strong evidence for topical repellents’ efficacy against mosquito biting [4], and some evidence of repellent effectiveness against malaria in clinical trials [5], the World Health Organization (WHO) has yet to endorse deployment of topical repellents for malaria prevention as an intervention with public health value [6]; importantly, no trial to our knowledge has investigated the effectiveness of the distribution of repellents in the context of large-scale disease prevention programmes in order to establish real-world effectiveness. If proven effective, incorporation of repellent into national strategies, particularly in areas where ITNs and IRS are less effective, may interrupt and reduce malaria transmission.

In the Greater Mekong Subregion (GMS), the emergence and spread of artemisinin-resistant malaria has accelerated the malaria elimination agenda, with GMS countries and WHO committing to eliminating malaria in the region by 2030. The overall approach includes providing universal access to malaria testing and treatment, and universal coverage of all at-risk populations with long-lasting insecticidal nets (LLINs) or IRS [7]. In GMS countries, village health volunteer (VHV) networks have been established to provide these malaria services to many villages, particularly where limited or no services exist (e.g., hard-to-reach groups/areas, conflict-affected/ceasefire areas). These national VHV networks provide important opportunities for implementing supplementary personal protection methods, such as repellents. Repellents may be highly relevant in the GMS because many malaria vectors readily feed outdoors and exhibit early biting behaviour [8], and groups that are at greater risk of exposure, namely those residing in or near forested areas, mobile workers, and migrant populations, have a specific need for personal protection [7].

In order to inform the rollout of repellent as part of a national strategic plan for malaria elimination, we conducted a stepped-wedge cluster randomised controlled trial to determine the effectiveness of the addition of repellent into the malaria services package delivered by the VHV network on reducing malaria infection in Myanmar. We examined the impact of repellent distribution on *Plasmodium falciparum* and *P*. *vivax* infection detected by rapid diagnostic test (RDT)—the routine method of malaria diagnosis in VHV networks in the GMS—as well as by polymerase chain reaction (PCR) (which exhibits higher sensitivity), and explored the moderating effect of intervention adherence and risk group on these associations. Among other reasons (outlined below), the stepped-wedge trial design was chosen to permit seminal analysis of the presence of delayed effects of repellent distribution as well as the impact of heterogeneity in malaria prevalence, at both a village and a temporal level, on the effectiveness of repellent in reducing malaria. Finally, a cost-effectiveness analysis of repellent distribution in the context of a large-scale malaria control and elimination programme was conducted in order to inform policy.

## Methods

### Ethics

This trial is registered in the Australian New Zealand Clinical Trials Registry (ACTRN12616001434482; approved retrospectively 14 October 2016) and was approved by the Ethics Review Committee on Medical Research involving Human Subjects, Department of Medical Research, Ministry of Health and Sports, Myanmar Government (#21/Ethics/2015; extended approval #Ethics/DMR/2016/020), and the Alfred Hospital, Melbourne, Australia (95/15). The ethics review committee of the Department of Medical Research, Ministry of Health and Sports, requested that there be no commercial advantage for the product during the trial. Consequently, the repellent was provided in plain unbranded tubes, and the investigators registered the trial after completion of fieldwork (but prior to the commencement of data analysis) to minimise public disclosure. The study protocol has been published previously [9], and the study is reported according to the CONSORT guidelines of reporting a stepped-wedge cluster randomised trial (S1 CONSORT Checklist).

### Trial design and randomisation

**Fig 1:**
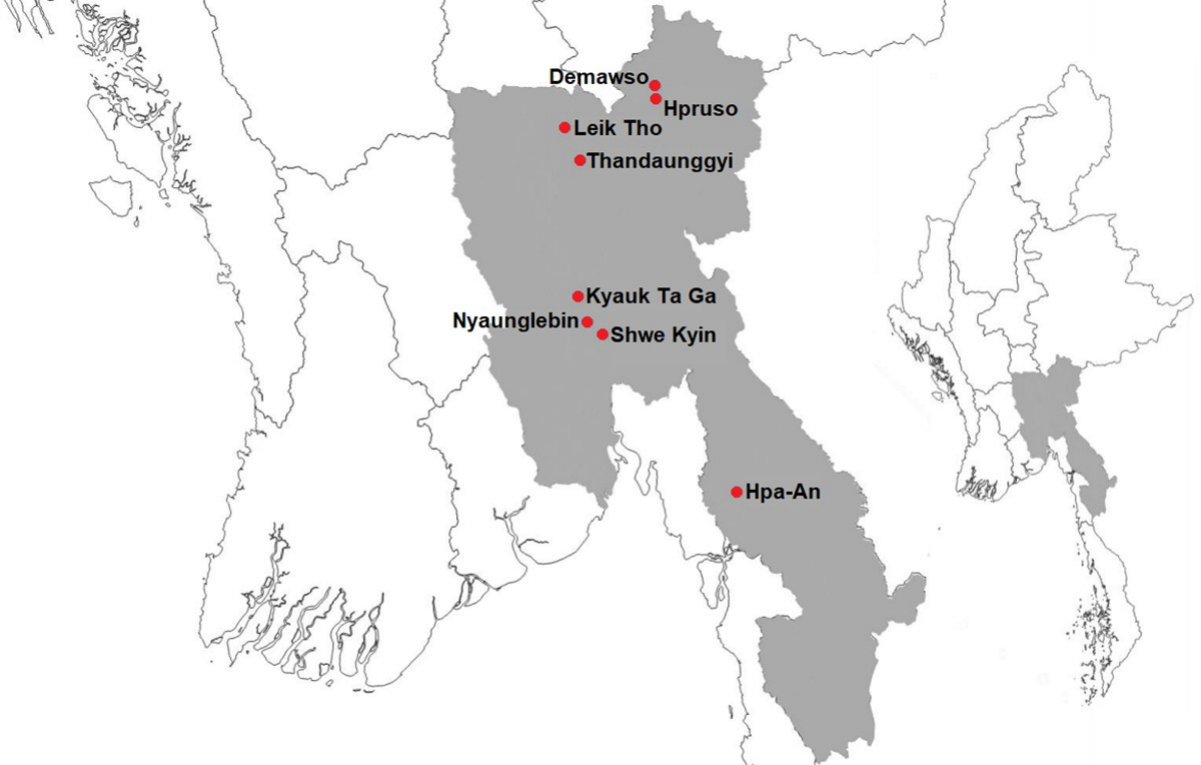
Location of townships selected for topical repellent trial participation in Kayin, Kayah, and Bago East states. Map was generated using the tmap package in R version 3.6.1 [10] with data from Natural Earth.

Between April 2015 and June 2016, a 15-month stepped-wedge cluster randomised trial was conducted in Myanmar with a target sample of 116 villages (clusters). Originally, in order to maximise power, we sought to confine data collection to the ‘rainy’ seasonal period only. However, due to logistical constraints in integrating research into implementation activities and in order to model temporal effects of repellent distribution, we conducted the study continuously through the ‘cool’, ‘hot’, and ‘rainy’ seasonal periods. Villages from 8 townships identified as having malaria services gaps according to National Malaria Control Programme services data were recruited, in Bago East (16 villages), Kayin (61 villages), and Kayah (39 villages) states (for map see Fig 1). All villages participated in LLIN distribution immediately prior to the start of the study. Using a computer-based block randomisation routine, the trial statistician randomised de-identified villages to respective ordered blocks to ensure the month at which villages started receiving the intervention during the 15-month study period was randomly determined. The design was such that after a minimum of 1 month without repellent distribution, blocks of 8 villages were sequentially stepped monthly (without transition period), from a no repellent state (control) into community-based repellent distribution by VHVs (intervention) until study end (12 villages transitioned in the final month, given the incomplete design). A cluster stepped-wedge design was implemented given the intervention necessitated implementation at the village level, the statistical performance of the design in terms of power (both within- and between-cluster variance is used), its capacity to model temporal effects, and the advantage of being able to deliver the intervention to all villages.

### Repellent intervention and implementation

The repellent distributed was 12% N,N-diethylbenzamide w/w cream because this product was the only licensed repellent for use in Myanmar at the time of the study and was readily available in the market to the study population. Results of laboratory evaluation and field trials showed no difference in percent protection against mosquito bites between 12% N,N-diethylbenzamide w/w cream and DEET cream (12% N, N-diethyl-3-methylbenzamide), a WHO-recommended positive control for testing the effectiveness of mosquito repellents [11,12]. Field trials of 12% N,N-diethylbenzamide w/w cream at 10 mg/cm^2^ dose showed 100% protection against 4 *Anopheles* species for up to 11 hours [11].

Repellent was distributed in 2 stages. In the first stage, repellent was introduced and distributed to all villagers (village residents, forest dwellers, and migrants) at village-level community meetings by VHVs and Karuna Mission Social Solidarity (KMSS) malaria officers (local field implementation staff who liaise between VHVs and implementing partners). Any village members absent from community meetings were identified by VHVs, and repellent was distributed to them individually, external to the meeting.

In the second stage, in order to establish and maintain consistent distribution, villagers were asked to return any empty repellent tubes to the VHV for replacement (although this was not a requirement for additional repellent distribution). The nature of the study and proper application of repellent was explained verbally (using a hardcopy guideline information sheet; S1 Text) to villagers by malaria officers during the initial community meeting and by VHVs in the process of distributing repellent outside of community meetings. Villagers were provided information regarding repellent composition, how repellent works to protect people from biting, proper application (including topics such as application sequencing, optimal amounts or volume, optimal timing of application, storage, and application to infants), risks of use, and contact information for reporting adverse effects and restocking supply. Villagers were asked to inform the VHV immediately if allergy or ingestion occurred. The VHVs checked the appropriate use of repellent by villagers when they returned to the VHV to replenish their repellent. The VHVs were required to do at least 1 mass community health education session per month, which was supervised by the malaria officers of KMSS. Repellent was provided in plain, unbranded tubes.

In order to determine the number of repellent tubes required to supply a village across the study, the respective village authority provided the village population size, and the number of tubes needed to be stocked was estimated by the in-country study team (2 tubes per person initially and an additional 20%–30% of this total allocation for the VHV-led repellent replacement). On a monthly basis, malaria officers communicated with VHVs regarding stock levels, with stock typically kept at 20%–30% of the initial procurement. Repellent was delivered to villages between 1 and 2 months prior to the onset of the randomly allocated distribution start month and stored in an appropriate secure location prior to commencement of the distribution process.

Household-level mapping was considered as a primary mode of repellent distribution; however, it was determined that village-level distribution through community meetings, with targeted follow-up of non-attenders would be the most pragmatic and cost-effective method of distribution.

### Outcome measures

#### Primary outcome

The primary outcome was *P*. *falciparum* or *P*. *vivax* (or both, collectively referred to as *Plasmodium* spp.) infection determined by SD Bioline P.f/P.v combo RDT, measured monthly by VHVs. This RDT is routinely used by VHVs in the field for active and passive case detection. Passive case detection refers to villagers presenting to VHVs for testing, and active case detection refers to VHVs seeking out infection in their village (e.g., during health education sessions or household visits) and performing a minimum number of tests set by the programme.

#### Secondary outcomes

Secondary outcomes included symptomatic malaria, defined as RDT positive plus fever and/or other malaria symptoms; PCR-detectable *Plasmodium* spp. infections; molecular markers of artemisinin resistance; and antimalarial antibody levels in individuals as determined by enzyme-linked immunosorbent assay [9]. In this paper we report data on PCR-detectable infections. Quantitative PCR was used to detect low-density *P*. *falciparum* and *P*. *vivax* infection in dried blood spots (S2 Text). All individuals presenting for routine RDT were offered the opportunity to provide a dried blood spot sample; these samples were collected by VHVs from individuals who provided written informed consent.

### Power

Power estimation was based on the estimation of an intervention effect from a stepped-wedge cluster randomised design assuming analysis by generalised linear mixed modelling (GLMM) [13]. Given the design, an estimate of malaria incidence of 1% (by RDT, based on unpublished data collected from villages during 2014), an expectation of testing approximately 20 participants for malaria using RDTs per month per village from 116 villages (over 15 months), and an estimate of between-village heterogeneity (intraclass correlation coefficient [ICC]) of 0.15, we estimated the study was powered to detect a 50% reduction in malaria infections due to topical repellent distribution with 90% power and 5% significance (2-sided) [9].

### Statistical analysis

GLMM was performed on individual-level observations to estimate the effect of repellent on *P*. *falciparum* or *P*. *vivax* (or both, (collectively referred to as *Plasmodium* spp.) infection (determined by RDT and PCR). Repellent was included as a binary variable that indicated a village’s repellent distribution status as being either without (i.e., control state) or with repellent (intervention state). This repellent variable was modelled as a time-varying (monotonic) variable whereby the variable changed in value from control to intervention for each village at a specific stage across the 15 months of the study. Models were generalised through use of a logit link function and binomial distribution (see S3 Text). A crossed random effects (or non-nested) framework was used to correctly account for dependencies in study participant probability of infection (i.e., participants from the same village were tested at different periods [months] and participants tested in a given period were from different villages), with random effects (i.e., intercepts) for both village (level 2) and month (level 2) crossed at the participant level (level 1). In addition to random effects for village and month (i.e., to model temporal clustering), the repellent intervention was also modelled as a time-varying random effect at the village level (specified a priori in the trial protocol). Inclusion of a random effect for repellent relaxes the assumption of a fixed, common effect of repellent across all villages in the GLMM, was achieved by estimating the effect of repellent at the village level (i.e., level 2) of the mixed model (i.e., multilevel model), and assumes village-specific differences in the effect of repellent (i.e., heterogeneity in effect caused by potential unmeasured factors such as adherence). In terms of intervention effect estimation, as the intervention was time-varying, both between-village (odds of malaria infection for villages in an intervention period compared to villages in a control period) and within-village (odds of malaria infection in intervention periods compared to control periods for a single village) effects are estimated in the GLMM, and the estimator is the weighted average of the between- and within-village estimators, where the weight for each estimator decreases as the standard error increases. The model also included fixed terms for estimating the effect of time (linear) and season (hot [March to April], rainy [May to October], and cool [November to February, reference group]) on malaria infection. Repellent distribution was modelled as an instantaneous factor (i.e., assumed to have an immediate effect at distribution) for the primary analysis and as a lagged factor in additional modelling (i.e., having a delayed effect of 1 and 2 months following distribution). This modelling framework and the composition of models (i.e., fixed and random components) were specified a priori and published in the study protocol (See S4 Text for the statistical analysis plan as described in the protocol) [9].

To explore the extent to which any association between repellent distribution and PCR-detectable infections varied across *Plasmodium* spp. (i.e., *P*. *falciparum* or *P*. *vivax*), generalised structural equation modelling (GSEM) was used to apply crossed random effects multinomial logit regression analyses. In this modelling, random effects for village and month were constrained to be equal across *Plasmodium* spp..

Given its direct impact on efficacy, we also performed an a priori specified additional analysis of the frequency of repellent application at the village level as the principal indicator of intervention implementation quality. At the end of the study, VHVs were asked to assess village frequency of repellent application (question: “In your village, on average, how often do you think community members used mosquito repellent cream?”) as occurring ‘never’, ‘monthly’, ‘weekly’, or ‘daily’ on average, and this variable was modelled in per protocol analysis. In addition, we explored the extent to which risk group membership (participant self-reported village resident, forest dweller, or migrant status) might moderate repellent effectiveness using a group by intervention fixed-effect interaction term in modelling.

ICCs were estimated for both village and temporal heterogeneity using estimated model variance components. Given the correct specification of the GLMM, the effect estimates for repellent distribution were unbiased in light of village attrition, assuming missing monthly RDT and PCR data were missing at random (MAR) [14].

Stata version 15.1 was used for all statistical analyses, randomisation, and power estimation [15].

### Cost-effectiveness analysis

We calculated the additional costs associated with adding repellent distribution to malaria services already programmatically funded and implemented by VHVs, excluding study-related costs. All costs are reported in 2015 US dollars and are given in S5 Text, as are methods for the cost-effectiveness analysis. The effectiveness outcome was PCR-detectable infections averted, and a sensitivity analysis was conducted using the upper and lower limits of the 95% reference range for village-specific malaria prevalence, which incorporated empirical Bayes random intercept predictions from GLMM.

## Results

### Study population, surveillance, and malaria prevalence

**Fig 2:**
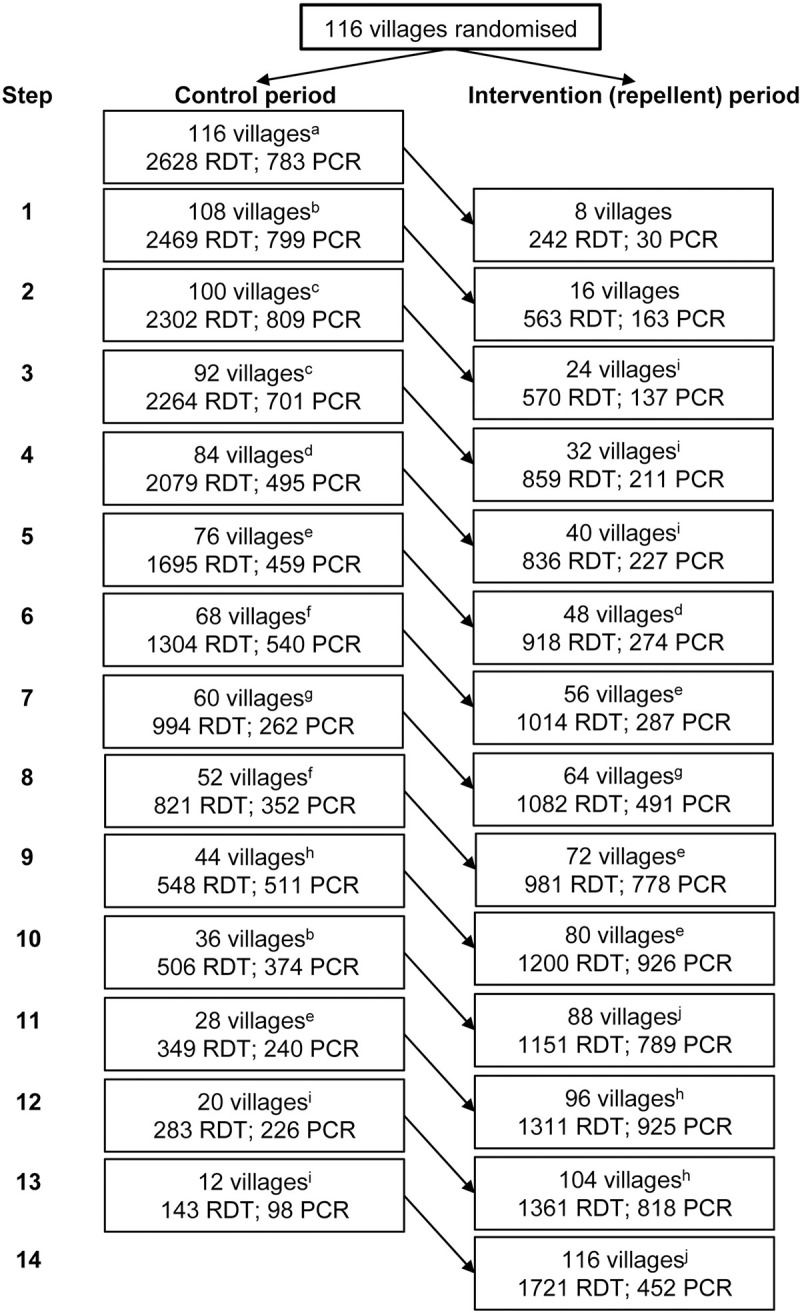
Flowchart showing the number of villages and number of tests observed by intervention period by step. The mean (SD) testing rate of RDTs was 20.5 (14.6) tests per village per month, and the mean (SD) number of months villages had observations for was 13.8 (1.8) months. For PCR, the mean (SD) monthly testing rate per village was 11.2 (7.9) tests, and the mean (SD) number of months villages had observations for was 10.6 (3.0). Missing village data for ^a^9, ^b^7, ^c^4, ^d^3, ^e^5, ^f^6, ^g^8, ^h^11, ^i^1, and ^j^12 villages including 2 villages that were enrolled and randomised into the trial but were unable to participate due to security reasons. PCR, polymerase chain reaction; RDT, rapid diagnostic test.

One hundred sixteen villages across 8 townships with an estimated total population of 31,016 were randomised to receive repellent. Of the original sample of 116 villages, 4 villages (3.4%) were excluded due to security concerns, with 2 villages substituted with alternate villages selected randomly. A total of 32,194 RDTs were performed across 114 villages during the 15-month study period (Fig 2). The mean (SD) testing rate of RDTs was 20.5 (14.6) tests per village per month, considering the mean (SD) number of months villages had observations for was 13.8 (1.8) months. The median age of participants undergoing rapid diagnostic testing was 18 years, and approximately half were female. Rapid diagnostic testing was performed on village residents (46%), forest dwellers (40%), and migrants (14%), with these risk groups similarly distributed across the control and intervention periods. *Plasmodium* spp. infection detected by RDT was lower than expected, with 50 infections (0.16%; 29 of which were symptomatic) detected over the duration of the trial (34 *P*. *vivax* mono-infections, 13 *P*. *falciparum* mono-infections, and 3 mixed-species infections; Table 1). A total of 13,157 dried blood spot samples were collected across 111 villages, with approximately 3% (419/13,157) of dried blood spot samples testing positive by PCR for *Plasmodium* spp. infections (123 *P*. *vivax* mono-infections, 207 *P*. *falciparum* mono-infections, and 89 mixed-species infections; Table 1). The mean (SD) testing rate of dried blood spot samples was 11.2 (7.9) per village per month, and the mean (SD) number of months villages had observations for was 10.6 (3.0) months.

**Table 1:** Participant characteristics and *Plasmodium* spp. infection status by control/intervention periods (*n =* 32,194 RDTs).

Participant characteristic	Control period (*n* = 18,385)	Intervention period (*n* = 13,809)
*Age*, *years*, *median [p25*, *p75]*	18 [9, 32]	18 [9, 33]
*Female*, *n (%)*	9,045 (49.2)	7,033 (50.9)
*Pregnancy*, *n (%)*	62 (0.69)	54 (0.77)
*Season*, *n (%)*		
Cool	2,869 (15.6)	4,277 (31.0)
Hot	3,260 (17.7)	2,462 (17.8)
Rainy	12,256 (66.7)	7,070 (51.2)
*Residential status*, n (%)*		
Migrant	2,817 (15.3)	1,598 (11.6)
Resident	8,733 (47.5)	6,050 (43.8)
Forest dweller	6,833 (37.2)	6,160 (44.6)
***Infection status*, *n (%)***		
*RDT*		
No infection	18,351 (99.8)	13,793 (99.9)
*P*. *falciparum*	9 (0.05)	4 (0.03)
*P*. *vivax*	23 (0.13)	11 (0.08)
Mixed species	2 (0.01)	1 (0.01)
Total infections	34 (0.18)	16 (0.12)
*PCR (n = 13*,*157)*		
No infection	6,390 (96.1)	6,348 (97.5)
*P*. *falciparum*	116 (1.7)	91 (1.4)
*P*. *vivax*	76 (1.1)	47 (0.7)
Mixed species	67 (1.0)	22 (0.3)
Total infections	259 (3.9)	160 (2.5)

*Three study participants missing residential status data.

p25, 25th percentile; p75, 75th percentile; PCR, polymerase chain reaction; RDT, rapid diagnostic test.

### The instantaneous association between repellent distribution and RDT- and PCR-detectable infections

**Fig 3:**
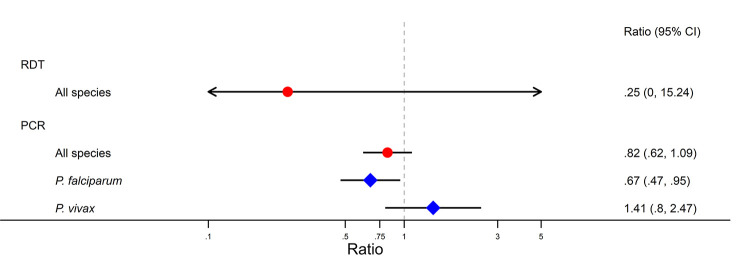
Forest plot showing the instantaneous association between village repellent distribution and *Plasmodium* spp. infection detected by RDT and PCR, including PCR species-specific analyses. Red circles indicate adjusted odds ratios; blue diamonds indicate adjusted relative risk ratios.

Rapid diagnostic testing is the method of malaria diagnosis routinely employed at the community level, and for RDT-detectable infections, we observed a non-significant reduction in the odds of *Plasmodium* spp. infection after repellent distribution by VHVs, independent of time and season (adjusted odds ratio [AOR] = 0.25, 95% CI 0.004–15.2, *p =* 0.512; Fig 3, Tables 2 and S1). When PCR-detectable infections were examined, repellent distribution was associated with an 18% reduction in the odds of any *Plasmodium* spp. infection (AOR = 0.82, 95% CI 0.62–1.09, *p =* 0.180) (Fig 3; Tables 2 and S2). Considering the impact of repellent distribution on species-specific infections, there was a 33% reduction in odds of *P*. *falciparum* infections (adjusted relative risk ratio [ARRR] = 0.67, 95% CI 0.47–0.95, *p =* 0.026) (Fig 3; Tables 2 and S3). However, there was no significant impact on *P*. *vivax* infections (ARRR = 1.41, 95% CI 0.80–2.47, *p =* 0.233). The difference in the impact of repellent across the 2 species was statistically significant (Wald χ^2^[1] = 5.24, *p =* 0.022). Although there were some differences in the impact of repellent on PCR-detectable infection across village residents, forest dwellers, and migrants, these differences were not statistically significant (*Plasmodium* spp. infection—resident: AOR = 0.65, 95% CI 0.44–0.96; forest dweller: AOR = 0.90, 95% CI 0.63–1.29; migrant: AOR = 1.13, 95% CI 0.62–2.06; Wald χ^2^[2] = 3.39, *p =* 0.183, estimated from the linear combination of the main effect of repellent distribution and the interaction between repellent distribution and resident status, see S4 Table; *P*. *falciparum—*resident: AOR = 0.58, 95% CI 0.38–0.88; forest dweller: AOR = 0.74, 95% CI 0.45–1.22; migrant: AOR = 0.77, 95% CI 0.35–1.72; *P*. *vivax* infection—resident: AOR = 1.17, 95% CI 0.58–2.36; forest dweller: AOR = 1.49, 95% CI 0.79–2.82; migrant: AOR = 2.05, 95% CI 0.70–5.95; joint Wald χ^2^[4] = 2.03, *p =* 0.730; See S5 Table).

**Table 2:** The instantaneous association between village repellent distribution and *Plasmodium* spp. infection detected by RDT and PCR.

Factor	RDT			PCR								
	All species* (*n* = 32,194)			All species (*n* = 13,157)			*P*. *falciparum*^†^ (*n* = 13,068)			*P*. *vivax*^†^ (*n* = 13,068)		
	AOR	95% CI	*p*-Value	AOR	95% CI	*p*-Value	ARRR	95% CI	*p*-Value	ARRR	95% CI	*p*-Value
*Intervention*												
No repellent	Ref.	—	—	Ref.	—	—	Ref.	—	—	Ref.	—	—
Repellent	0.25	0.004, 15.2	0.512	0.82	0.62, 1.09	0.180	0.67	0.47, 0.95	0.026	1.41	0.80, 2.47	0.233
*Time (month)*	0.87	0.72, 0.97	0.013	0.97	0.89, 1.07	0.582	1.02	0.95, 1.11	0.523	0.95	0.87, 1.04	0.247
*Season*												
Cool	Ref.	—	—	Ref.	—	—	Ref.	—	—	Ref.	—	—
Hot	3.64	0.89, 14.8	0.072	1.10	0.35, 3.44	0.871	0.81	0.27, 2.40	0.700	11.2	2.23, 56.2	0.003
Rainy	3.15	0.89, 11.1	0.074	1.17	0.45, 3.02	0.747	0.46	0.17, 1.21	0.115	20.5	4.84, 86.6	<0.001

Instantaneous treatment effect comparisons: AORs and ARRRs, 95% CIs, and *p*-values from generalised linear mixed modelling (GLMM) using generalised structural equation modelling (GSEM).

*All-species estimates from crossed random effects generalised (logit) linear mixed model with random effects for cross-sectional (month) and village-specific heterogeneity in infection, and village-specific heterogeneity in effect of repellent distribution.

^**†**^Species-specific (ARRR) estimates from crossed random effects generalised (multinomial) linear mixed model with random effects for cross-sectional (month) and village-specific heterogeneity in infection. No infection was the reference group for the outcome.

AOR, adjusted odds ratio; ARRR, adjusted relative risk ratio; PCR, polymerase chain reaction; RDT, rapid diagnostic test.

### Heterogeneity in *Plasmodium* spp. infection and the impact of repellent distribution

**Fig 4:**
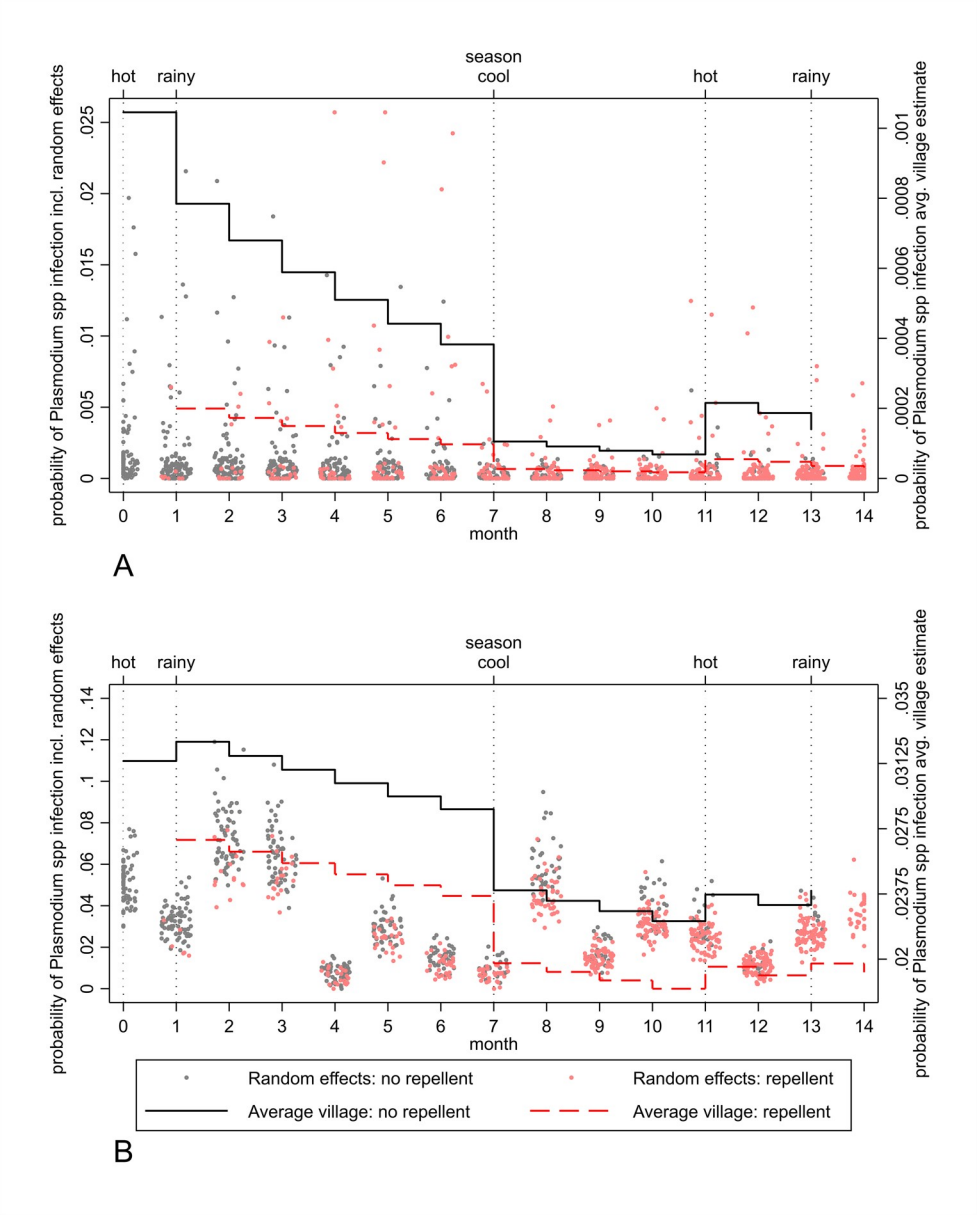
Overall and village-specific probabilities of *Plasmodium* spp. infection detected by RDT and PCR by month, season, and repellent status. *Plasmodium* spp. infection detected by (A) RDT and (B) PCR. Figure shows differences in probability (i.e., model-based prevalence) of *Plasmodium* spp. infection by time, season, and intervention status from generalised linear mixed modelling (GLMM) on average (lines). In addition, it shows Bayes random effect predictions of village-specific probabilities of infection (dots) by the same factors. In terms of heterogeneity, the figure demonstrates the higher level of heterogeneity in infection between villages on average (dispersion of red and black dots considered together) and when in the intervention phase as compared to the control phase (dispersion of red dots versus black dots), for infections detected by RDT compared to PCR. PCR, polymerase chain reaction; RDT, rapid diagnostic test.

To determine whether the apparent effectiveness of repellent was influenced by variation in the level of infection in villages and over time, and to describe trial results graphically, post hoc estimates of the probability of infection detected by RDT (Fig 4A) and PCR (Fig 4B) at the village level (which include random effects), as well as the estimated average probability for intervention and control periods, were produced from the GLMM. For RDT-detectable *Plasmodium* spp. infection, there was marked heterogeneity (as indicated by the estimated probabilities that include village- and time-specific random effects in Fig 4A): In several villages the risk of *Plasmodium* spp. infection was significantly higher than in the majority, and the risk of infection greater during intervention (ICC = 0.75) compared to control periods (ICC = 0.42) (see S1 and S2 Tables and S6 Text for information regarding ICCs and tests of inference for random terms). We observed very little temporal (i.e., between-month) heterogeneity in *Plasmodium* spp. infection detected by RDT (ICC = 0.003). However, we did observe higher temporal heterogeneity in the probability of *Plasmodium* spp. infection detected by PCR (ICC = 0.13) (Fig 4B). For PCR-detectable *Plasmodium* spp. infection, there was a lower level of village-specific heterogeneity (ICC = 0.03) and no village-specific differences in the association between repellent and infection (Fig 4B; S6 Text). The low heterogeneity of the effect of repellent on PCR-detectable infections between villages suggests that repellent as an intervention to reduce malaria is likely to be applicable across a range of transmission settings.

### The delayed association between repellent distribution and RDT- and PCR-detectable infections

To explore the extent to which the effect of the rollout of repellent on *Plasmodium* spp. infection was time-dependent, we modelled the impact of repellent distribution on *Plasmodium* spp. infection delayed by 1 and 2 months after distribution (Tables 3, S1, S2 and S6; S1 Fig). For RDT-detectable infections, delayed effects were estimated to be markedly stronger (1 month: AOR = 0.14, 95% CI 0.003–7.74, *p =* 0.336; 2 months: AOR = 0.01, 95% CI 0.0001–0.77, *p =* 0.038) than when assuming that the impact of repellent distribution occurred instantaneously (Table 3). Although not as marked as for *Plasmodium* spp. infection detected by RDT, analyses of time dependence for PCR-detectable infections showed apparent delayed effects that were estimated to be stronger than assuming an instantaneous effect (1 month: AOR = 0.77, 95% CI 0.58–1.03, *p =* 0.080; 2 months: AOR = 0.75, 95% CI 0.55–1.01, *p =* 0.062). The time dependence of the effect of repellent distribution was less consistent for species-specific analyses (Tables 3 and S6; S1 Fig) than when assuming an instantaneous impact.

**Table 3:** The delayed impact of village repellent distribution on *Plasmodium* spp. infection detected by RDT and PCR.

Factor	RDT			PCR								
	All species* (*n* = 32,194)			All species (*n* = 13,157)			*P*. *falciparum*^†^ (*n* = 13,068)			*P*. *vivax*^†^ (*n* = 13,068)		
	AOR	95% CI	*p*-Value	AOR	95% CI	*p*-Value	ARRR	95% CI	*p*-Value	ARRR	95% CI	*p*-Value
***1-month delay***												
*Intervention*												
No repellent	Ref.	—	—	Ref.	—	—	Ref.	—	—	Ref.	—	—
Repellent	0.14	0.003, 7.74	0.336	0.77	0.58, 1.03	0.080	0.77	0.53, 1.11	0.163	0.97	0.57, 1.65	0.910
*Time (month)*	0.84	0.76, 0.94	0.003	0.98	0.89, 1.07	0.631	1.01	0.92, 1.12	0.785	0.97	0.89, 1.06	0.511
*Season*												
Cool	Ref.	—	—	Ref.	—	—	Ref.	—	—	Ref.	—	—
Hot	3.80	0.96, 15.1	0.058	1.11	0.36, 3.47	0.856	0.81	0.29, 2.26	0.688	11.0	3.44, 35.5	<0.001
Rainy	3.49	1.00, 12.1	0.049	1.18	0.46, 3.03	0.738	0.46	0.22, 0.96	0.039	20.2	8.56, 47.5	<0.001
***2-month delay***												
*Intervention*												
No repellent	Ref.	—	—	Ref.	—	—	Ref.	—	—	Ref.	—	—
Repellent	0.01	0.0001, 0.77	0.038	0.75	0.55, 1.01	0.062	0.81	0.55, 1.17	0.252	0.77	0.40, 1.50	0.444
*Time (month)*	0.87	0.77, 0.97	0.016	0.98	0.89, 1.07	0.634	1.01	0.92, 1.11	0.846	0.99	0.88, 1.10	0.796
*Season*												
Cool	Ref.	—	—	Ref.	—	—	Ref.	—	—	Ref.	—	—
Hot	3.72	0.93, 14.9	0.064	1.12	0.36, 3.49	0.850	0.81	0.24, 2.70	0.732	11.2	2.1, 60.8	<0.001
Rainy	3.89	1.09, 13.9	0.036	1.18	0.46, 3.05	0.731	0.46	0.17, 1.22	0.120	20.3	4.74, 86.9	<0.001

Delayed treatment effect comparisons: AORs and ARRRs, 95% CIs, and *p*-values from generalised linear mixed modelling (GLMM).

*All-species estimates from crossed random effects generalised (logit) linear mixed model with random effects for cross-sectional (month) and village-specific heterogeneity in infection, and village-specific heterogeneity in effect of repellent distribution.

^**†**^Species-specific (ARRR) estimates from crossed random effects generalised (multinomial) linear mixed model with random effects for cross-sectional (month) and village-specific heterogeneity in infection. No infection was the reference group for the outcome.

AOR, adjusted odds ratio; ARRR, adjusted relative risk ratio; PCR, polymerase chain reaction; RDT, rapid diagnostic test.

### Analysis of adherence to intervention (per protocol analysis)

During the study there were no reports of allergy or ingestion of the repellent, which was a publicly available and locally approved product for which efficacy and adverse outcomes had been previously established. To examine the impact of frequency of repellent application on repellent effectiveness, VHVs determined the frequency of repellent use in their village. Complete adherence to the intervention varied; just over half of the VHVs (56%, 55/98) reported that, on average, study participants applied repellent ‘daily’ and that the majority (67%, 68/102) also applied the repellent as per instructions. Six villages (6%, 6/102) experienced repellent stock-out periods. In these analyses, the intervention was measured as an ordinal time-varying exposure, where villages could be in either a control or an intervention state, which was marked by ‘monthly’, ‘weekly’, or ‘daily’ average use. Although these analyses (S7 Table) showed that overall exposure to the repellent intervention was not statistically significant (joint Wald χ^2^[3] = 3.45, *p =* 0.328), there was a descriptive pattern of lower risk of *Plasmodium* spp. infection detected by RDT with higher levels of VHV-reported frequency of repellent use (repellent used monthly: AOR = 1.54, 95% CI 0.14–16.7; repellent used weekly: AOR = 0.33, 95% CI 0.01–22.2; repellent used daily: AOR = 0.05, 95% CI 0.002–10.3). A similar descriptive pattern was observed for malaria infection detected by PCR across levels of exposure, but again this was not statistically significant (joint Wald χ^2^[3] = 4.7, *p =* 0.195) (S8 Table).

### Cost-effectiveness of repellent

The total cost of the intervention during the study was US$76,138 (tubes of repellent, US$28,139; staff costs, US$45,172; rent, US$1,257; distribution, US$1,524; meetings, US$47). In total, repellent was provided for 237,701 person-months, or 19,808 person-years, resulting in a cost of approximately US$3.8 per person per year. The control provision (i.e., usual care) was provided for a total of 215,675 person-months, or 17,973 person-years.

Cost-effectiveness analysis based on the overall infection prevalence observed during the study (Table 4) found that the anticipated costs for a cohort of 10,000 people would be $US38,437 per year while averting 150 infections detected by PCR. This resulted in an incremental cost-effectiveness ratio (ICER) of US$256 per PCR-detected infection averted from repellent distribution. The sensitivity analysis applying the 95% reference range for village-specific malaria prevalence resulted in 182 cases averted for the upper and 46 cases averted for the lower PCR limits, corresponding to ICERs of $US212 and US$832, respectively.

**Table 4:** Cost-effectiveness results for a population of 10,000 people over 1 year (costs are in US dollars).

Measure	Intervention	
	Usual care	Usual care + repellent
Incremental costs	—	US$38,437
Cases detected by PCR	400	250
PCR infections averted	—	150
ICER per PCR-detected infection averted	—	US$256

ICER, incremental cost-effectiveness ratio; PCR, polymerase chain reaction.

## Discussion

The results from this trial demonstrated that, although incorporation of repellent delivery into VHV-delivered malaria services did not reduce RDT-detected *Plasmodium* spp. infections, apparent protection was observed against PCR-detected *P*. *falciparum*, but not *P*. *vivax*, infection. Importantly, for PCR-detectable infections, there was low heterogeneity of this protective effect between villages, and the effect was similar between village residents and migrants/forest workers, suggesting that repellent as an intervention to reduce malaria is likely to be applicable across a range of transmission settings and populations. Large-scale distribution of repellent delivered through VHVs, or similar providers, as part of malaria control and elimination programmes may be an effective public health strategy to target residual malaria transmission not covered by conventional vector control measures in Myanmar and the GMS more broadly.

One of the barriers to including or recommending repellents for malaria prevention is the lack of evidence on implementation strategies that are effective. To our knowledge, this trial is the first to evaluate topical repellent distribution as part of a broader public health campaign, establishing evidence for the effectiveness of repellent distributed by the network of VHVs who deliver endorsed malaria interventions in Myanmar and the GMS more broadly. Our findings suggest that implementation using the VHV network model, or similar infrastructures, could be a mechanism for providing repellents for malaria prevention at the required scale. We found that the impact of repellent was species-specific; repellent distribution significantly reduced PCR-detectable *P*. *falciparum*, but not *P*. *vivax*, infections, and this could be seen as providing discriminant validity for repellent effectiveness. The limited effect on *P*. *vivax* is most likely explained by the large proportion of *P*. *vivax* infections that are caused by relapses from dormant liver stages, rather than being new infections acquired from mosquito bites, whereas *P*. *falciparum* does not have a dormant liver stage. This strengthens the internal validity of our study because it suggests that repellent can specifically protect against new *Plasmodium* spp. infections. Whether the density of a new *Plasmodium* spp. infection reaches the detection limits of routine RDTs or PCR is dependent on the level of naturally acquired immunity, which controls parasitaemia. RDT-negative, PCR-detectable infections are important in malaria elimination settings because they often go undetected and untreated and can contribute to ongoing malaria transmission [16]. This is particularly important in the GMS, where there is an urgent need to interrupt the transmission of artemisinin-resistant *P*. *falciparum*. Studies in the GMS have shown that PCR-detectable infections can harbour transmissible gametocytes, and increases in PCR-detectable infections also increase the entomological inoculation rate [16–18]. Furthermore, the density of PCR-detectable infections can change over time, and such infections eventually become detectable by less sensitive conventional diagnostics such as RDTs [19,20]. Therefore, the finding that repellent may also reduce the infectious reservoir strengthens the use of this intervention in malaria elimination settings in the GMS.

Interpretation of the effectiveness of repellent from trials in low transmission areas is challenging because insufficient power may result from low event rates, compounded by the low sensitivity of conventional malaria parasite detection methods. Previous trials in low transmission areas (<1% and <10% by RDT and microscopy, respectively), which did not integrate repellent into established health systems, reported that repellents were not associated with reductions in RDT-detectable infections (hazard ratio = 1.00, 95% CI 0.99–1.02) [21], but could reduce microscopically detectable *P*. *falciparum* infections by 28% to 82%, with varying degrees of statistical significance [22–25]. Only 1 other trial, also conducted in the GMS, has examined the effect of repellent distribution on PCR-detectable *P*. *falciparum* infections (<5% prevalence), and the researchers determined that their study was underpowered to detect the magnitude of effect observed, but confidence intervals did contain the magnitude of the apparent protective effect we observed (AOR = 0.83, 95% CI 0.44–1.56) [26]. Our trial was also limited by the lower than expected number of infections observed during the study period, which adversely affected the power to reliably provide inference with respect to the study’s a priori hypothesised effect of repellent distribution on *Plasmodium* spp. infection detected by RDT, the routine method of malaria diagnosis in the GMS. Despite this, the greater sensitivity of the secondary outcome measure of *Plasmodium* spp. infection detected by PCR, which yielded 8 times the number of events, combined with our study design and analytical approach, permitted effect estimation for repellent distribution with markedly greater accuracy in terms of inference.

A strength of our trial, compared to previous trials, was the stepped-wedge design, whereby village *Plasmodium* spp. infections are observed repeatedly and frequently in both the control and intervention states (as opposed to serial cross-sectional surveys). This repeat sampling frequency allowed us to explore delayed effects of repellent distribution—no delayed effect was found for PCR-detectable infections, but a 2-month delayed effect for RDT-detectable infections was present—and permitted both within- and between-village differences to be incorporated into statistical modelling. The estimation of crossed random effects (in addition to explicitly controlling for time and seasonality through fixed effects) permitted more effective partitioning of any variance in risk of infection from unmeasured factors impacting at both temporal and village-specific levels, resulting in increased precision of the average independent effect of repellent distribution. In addition, it enabled the quantification of heterogeneity in both village-specific baseline and time-specific risk of infection, as well as village-specific heterogeneity in the treatment effect. Interestingly, the effect of repellent on reducing PCR-detectable infections was consistent across villages, suggesting that repellent distributed by VHVs may be an effective intervention across a range of transmission settings and populations. Further studies are warranted across different transmission settings and populations, from the GMS and beyond, in order to inform WHO public health policy on the deployment of topical repellents for malaria prevention [6].

As an effectiveness trial, this study applied a principally ‘intention to treat’ approach by design, measuring the effect of repellent distribution by VHVs on malaria at a village level. Participants were not obliged to use repellent, and, given the population size, compliance and the usage of repellent by individuals was not strictly monitored. However, analysis based on VHVs’ estimates of the average frequency of repellent use in their village indicated lower risk of *Plasmodium* spp. infection where daily or weekly use (compared to monthly) was observed—but it should be noted the effect here was not statistically significant. Differences in compliance rates may explain the poor effectiveness of repellents when translated into field settings—possibly indicated by heterogeneity in levels of effectiveness across trials [5]. Whether the apparent protective effect of repellent observed in our trial was due to higher uptake and compliance is unknown; reported details of repellent messaging are limited in other trials undertaken in the GMS [21,25,26]. Nonetheless incorporation of repellent into public health programmes should be coupled with specific messaging pertaining to the importance of proper and consistent use of this preventive treatment.

There were limitations to the findings of this study. As mentioned previously, inference for the primary outcome of our study (the a priori hypothesised effect of repellent distribution on *Plasmodium* spp. infection detected by RDT) was limited by the lower than expected number of infections observed during the study period. Despite this, the greater sensitivity of the secondary outcome measure (*Plasmodium* spp. infection detected by PCR) and the subsequent higher number of events permitted modelling with increased precision for the estimate of the intervention effect. The geographical proximity of some villages may have resulted in contamination of some villages with repellent while in the control state. If this did occur, then this would bias results towards the null, meaning that the true protective effect of repellent may be larger than we observed. In the village, repellent was made available to high-risk groups (migrants and forest dwellers) as well as village residents, and we found that these groups were balanced in control and intervention periods and that the protective effect of repellent was similar across these groups. More targeted distribution campaigns for high-risk groups in areas where high-risk groups harbour the vast majority of infections may be beneficial because individuals in these high-risk groups are less likely to use ITNs or LLINs than the general population [27]. Different repellents should also be investigated. In our trial we used the only repellent available for use in Myanmar, but as additional products become available/endorsed by WHO they will also require assessment. The study design employed in this trial, together with reported ICCs, provides an important framework for designing and testing other malaria interventions in the GMS.

In our trial it cost US$3.8 per person per year to distribute repellent, and there was an ICER of US$256 to avert a PCR-detectable infection. The cost estimate is conservative because many households would still have had supplies when the study period ended and the cost savings due to reductions in healthcare use were not included. As areas move towards malaria elimination, the ICER for all malaria interventions will increase. Accordingly, we provided ICERs for the range of village-specific malaria prevalences observed in our study. These figures can inform the allocative-efficiency analyses of national malaria control programmes, maximising the impact of malaria interventions to achieve malaria elimination goals.

Establishing the effectiveness of repellents and distribution models for their implementation is essential in order to inform the broadening toolbox for the malaria elimination agenda, particularly in regions where other vector control interventions are losing efficacy. The findings of this stepped-wedge cluster randomised controlled trial, where the design and statistical modelling advance on previous research, suggest that integrating repellent into the package of malaria services provided by the VHV network may be an effective supplementary intervention for reducing new *Plasmodium* spp. infections in hard-to-reach villages of the GMS. Incorporation of repellent into national malaria control programme national strategic plans for malaria elimination may advance achieving GMS malaria elimination targets of 2025 for *P*. *falciparum* and 2030 for all malaria species. Furthermore, this strategy for repellent implementation could be suitable in other malaria-endemic settings globally.

## Supporting information

S1 CONSORT Checklist (PDF)

S1 Fig Forest plot showing delayed repellent effects—RDT-detectable infections, PCR-detectable infections, and species-specific PCR-detectable infections. Red circles indicate adjusted odds ratios; blue diamonds indicate adjusted relative risk ratios.
(TIF)

S1 Table The effect of village repellent distribution on *Plasmodium* spp. infection detected by RDT—instantaneous and delayed treatment effect comparisons. (DOCX)

S2 Table The effect of village repellent distribution on *Plasmodium* spp. infection detected by PCR—instantaneous and delayed treatment effect comparisons. (DOCX)

S3 Table The instantaneous effect of village repellent distribution on *P*. *falciparum* and *P*. *vivax* infection detected by PCR. (DOCX)

S4 Table Analysis of moderating effect of residency status: PCR-detected *Plasmodium* spp. infection and repellent distribution moderated by participant residency status. (DOCX)

S5 Table Analysis of the moderating effect of resident status: *P*. *falciparum* and *P*. *vivax* infection and repellent distribution moderated by participant resident status. (DOCX)

S6 Table The delayed effect of village repellent distribution on *P*. *falciparum* and *P*. *vivax* infection detected by PCR. (DOCX)

S7 Table Analysis of intervention adherence effects (per protocol analysis): RDT-detected *Plasmodium* spp. infection and repellent distribution incorporating VHV assessment of village-specific frequency of use. (DOCX)

S8 Table Analysis of adherence to intervention (per protocol analysis): PCR-detected *Plasmodium* spp. infection and repellent distribution incorporating VHV assessment of village-specific frequency of use. (DOCX)

S1 Text Guidelines for repellent usage. (DOCX)

S2 Text Molecular determination of *Plasmodium* spp. infections. (DOCX)

S3 Text Statistical notation for the generalised linear mixed model used to estimate the effect of repellent distribution on *Plasmodium* spp. infection. (DOCX)

S4 Text Statistical analysis plan. (DOCX)

S5 Text Repellent costs. (DOCX)

S6 Text Results of inferential tests assessing heterogeneity in the effect of repellent distribution using GLMM. (DOCX)

## Abbreviations

AOR: adjusted odds ratio
ARRR: adjusted relative risk ratio
GLMM: generalised linear mixed modelling
GMS: Greater Mekong Subregion
ICC: intraclass correlation coefficient
ICER: incremental cost-effectiveness ratio
IRS: indoor residual spraying
ITN: insecticide-treated bed net
KMSS: Karuna Mission Social Solidarity
LLIN: long-lasting insecticidal net
PCR: polymerase chain reaction
RDT: rapid diagnostic test
VHV: village health volunteer
WHO: World Health Organization
