# Supplementary figures and images for: Evaluation of the effectiveness of topical repellent distributed by village health volunteer networks against Plasmodium spp. infection in Myanmar: A stepped-wedge cluster randomised trial
Source: PLoS Med. 2020 Aug 20;17(8):e1003177. doi: 10.1371/journal.pmed.1003177 (PMC7444540; doi:10.1371/journal.pmed.1003177)

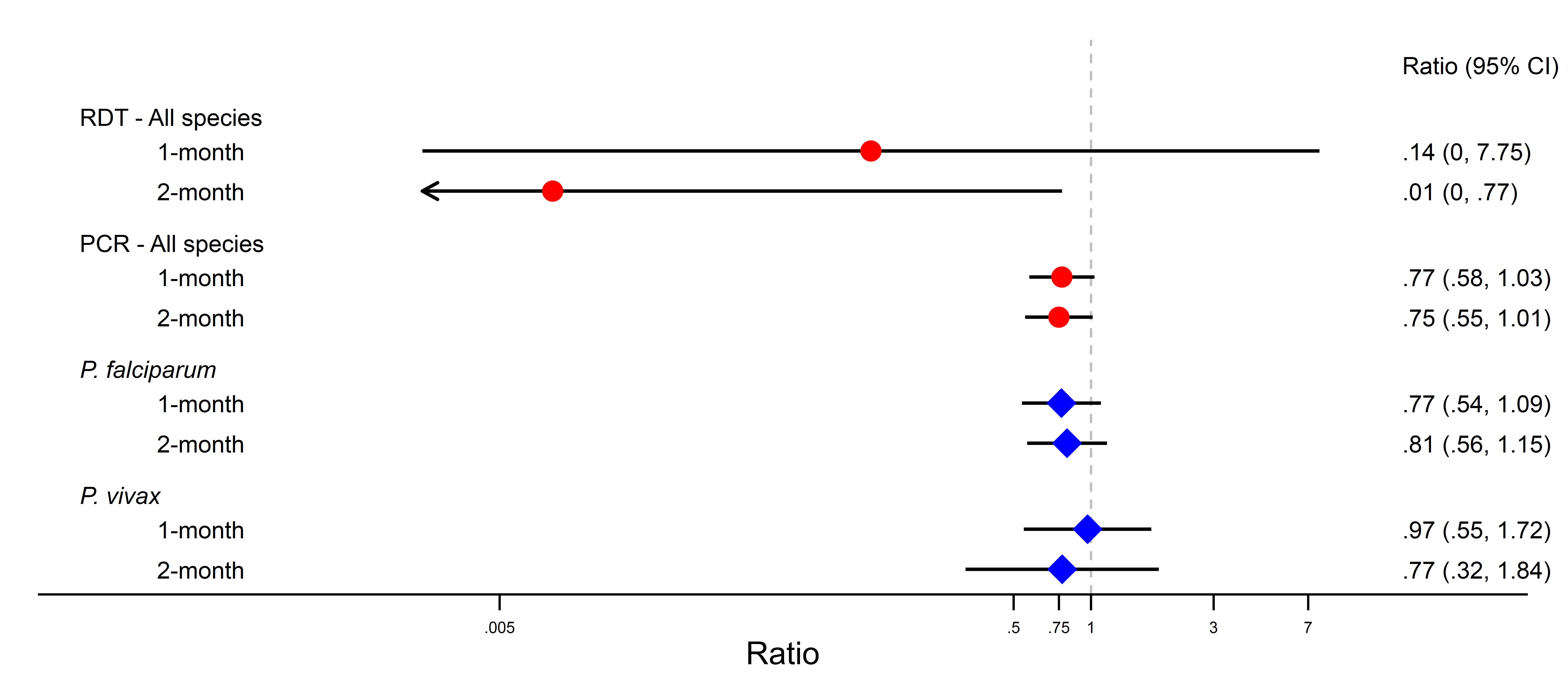

Supplement: S1 Fig — Red circles indicate adjusted odds ratios; blue diamonds indicate adjusted relative risk ratios. (TIF) [file pmed.1003177.s002.tif]
