# Supplementary material for: Evaluation of the effectiveness of topical repellent distributed by village health volunteer networks against Plasmodium spp. infection in Myanmar: A stepped-wedge cluster randomised trial
Source: PLoS Med. 2020 Aug 20;17(8):e1003177. doi: 10.1371/journal.pmed.1003177 (PMC7444540; doi:10.1371/journal.pmed.1003177)
Supplement: S1 Table — (DOCX) [file pmed.1003177.s003.docx]

S1 Table. The effect of village repellent distribution on *Plasmodium* spp. infection by using rapid diagnostic test (RDT): instantaneous and delayed treatment effect comparisons (n=32,194 RDTs performed over 15 months)

|  | | ***Instantaneous*** | | | |  | ***1-month delay*** | | | |  | ***2-month delay*** | | | |
| --- | --- | --- | --- | --- | --- | --- | --- | --- | --- | --- | --- | --- | --- | --- | --- |
| **Factors** | | **AOR** | ***95% CI*** | ***p-value*** | ***RE*** |  | ***AOR*** | ***95% CI*** | ***p-value*** | ***RE*** |  | ***AOR*** | ***95% CI*** | ***p-value*** | ***RE*** |
|  | |  |  |  |  |  |  |  |  |  |  |  |  |  |  |
| ***Fixed component*** | |  |  |  |  |  |  |  |  |  |  |  |  |  |  |
|  | |  |  |  |  |  |  |  |  |  |  |  |  |  |  |
| *Intervention* | |  |  |  |  |  |  |  |  |  |  |  |  |  |  |
|  | No repellent | ref. | - | - | - |  | ref. | - | - | - |  | ref. | - | - | - |
|  | Repellent | 0.25 | 0.004,15.2 | 0.512 | - |  | 0.14 | 0.003,7.74 | 0.336 | - |  | 0.01 | 0.0001,0.77 | 0.038 | - |
|  | |  |  |  |  |  |  |  |  |  |  |  |  |  |  |
| *Time (month)* | | 0.87 | 0.72,0.97 | 0.013 | - |  | 0.84 | 0.76,0.94 | 0.003 | - |  | 0.87 | 0.77,0.97 | 0.016 | - |
|  | |  |  |  |  |  |  |  |  |  |  |  |  |  |  |
| *Season* | |  |  |  |  |  |  |  |  |  |  |  |  |  |  |
|  | Cool | ref. | - | - | - |  | ref. | - | - | - |  |  |  |  | - |
|  | Hot | 3.64 | 0.89,14.8 | 0.072 | - |  | 3.80 | 0.96,15.1 | 0.058 | - |  | 3.72 | 0.93,14.9 | 0.064 | - |
|  | Rainy | 3.15 | 0.89,11.1 | 0.074 | - |  | 3.49 | 1.00,12.1 | 0.049 | - |  | 3.89 | 1.09,13.9 | 0.036 | - |
|  | |  |  |  |  |  |  |  |  |  |  |  |  |  |  |
| ***Random component*** | |  |  |  |  |  |  |  |  |  |  |  |  |  |  |
|  | |  |  |  |  |  |  |  |  |  |  |  |  |  |  |
| $\psi_{1}$^c^ | |  |  |  | 0.02 |  |  |  |  | - |  |  |  |  | 0.02 |
| $\psi_{2}$ | |  |  |  | 2.45 |  |  |  |  | 2.97 |  |  |  |  | 2.77 |
| $\psi_{3}$ | |  |  |  | 7.58 |  |  |  |  | 16.8 |  |  |  |  | 52.9 |
| $\rho_{01}$^d^ | |  |  |  | 0.42 |  |  |  |  | 0.47 |  |  |  |  | 0.46 |
| $\rho_{02}$^e^ | |  |  |  | 0.43 |  |  |  |  | 0.47 |  |  |  |  | 0.46 |
| $\rho_{11}$^f^ | |  |  |  | 0.75 |  |  |  |  | 0.86 |  |  |  |  | 0.94 |
| $\rho_{12}$^g^ | |  |  |  | 0.75 |  |  |  |  | 0.86 |  |  |  |  | 0.94 |
| $\rho_{2}$^h^ | |  |  |  | 0.003 |  |  |  |  | - |  |  |  |  | .003 |
|  | |  |  |  | *-344.12* |  |  |  |  | -340.5 |  |  |  |  | -339.1 |
|  | |  |  |  |  |  |  |  |  |  |  |  |  |  |  |
| ***Model fit indices*** | |  |  |  |  |  |  |  |  |  |  |  |  |  |  |
| *AIC* | |  |  |  | 704.2 |  |  |  |  | 697.1 |  |  |  |  | 694.2 |
| *BIC* | |  |  |  | 771.3 |  |  |  |  | 764.1 |  |  |  |  | 761.3 |

Instantaneous and delayed treatment effect comparisons: adjusted odds ratio (AOR), 95% confidence interval (95% CI), probability value (p-value), random-effect variances ($\psi$), conditional intraclass correlation coefficient ($\rho$)^a^ and model log likelihood () from generalised linear mixed modelling (GLMM)^b^

^a^ *ρ* = $\frac{\psi_{k}+ ...+ \psi_{nk}}{\psi_{k}+ ...+ \psi_{nk}+ {\pi^{2}}/3}$ , where $\psi_{k}$ through $\psi_{nk}$ are random-effect (RE) variance estimates pertaining to each of the respective crossed-classified variance components (see table notes ^c-h^) from the crossed random–effect generalised (logit) linear mixed models for a specific ICC estimate.

^b^ Crossed random-effect generalised (logit) linear mixed model with random-effects for temporal-specific (month), village-specific heterogeneity in infection and village-specific heterogeneity in effect of repellent distribution. Likelihood ratio tests did not support a less-constrained model whereby a covariance term for village-specific probability of Plasmodium infection and heterogeneity in intervention effect was estimated.

^c^$\psi_{1}$, $\psi_{2}$ and $\psi_{3}$ represent variances of the random-effects for month, village and intervention respectively.

^d^$\rho_{01}$ represents conditional ICC for participant tests conducted in the same village but different month in a control period.

^e^$\rho_{02}$represents conditional ICC for participant tests conducted in the same village and same month in a control period.

^f^$\rho_{11}$ represents conditional ICC for participant tests conducted in the same village but different month in an intervention period.

^g^$\rho_{12}$ represents conditional ICC for participant tests conducted in the same village and same month in an intervention period.

^h^$\rho_{2}$ represents conditional ICC for participant tests in the same month.
