# Supplementary material for: Evaluation of the effectiveness of topical repellent distributed by village health volunteer networks against Plasmodium spp. infection in Myanmar: A stepped-wedge cluster randomised trial
Source: PLoS Med. 2020 Aug 20;17(8):e1003177. doi: 10.1371/journal.pmed.1003177 (PMC7444540; doi:10.1371/journal.pmed.1003177)
Supplement: S2 Table — (DOCX) [file pmed.1003177.s004.docx]

S2 Table. The effect of village repellent distribution on *Plasmodium* spp. infection using Polymerase Chain Reaction (PCR) detection: instantaneous and delayed treatment effect comparisons (n=13,157)

|  | | ***Instantaneous*** | | | |  | ***1-month delay*** | | | |  | ***2-month delay*** | | | |
| --- | --- | --- | --- | --- | --- | --- | --- | --- | --- | --- | --- | --- | --- | --- | --- |
| **Factors** | | **AOR** | ***95% CI*** | ***p-value*** | ***RE*** |  | **AOR** | ***95% CI*** | ***p-value*** | ***RE*** |  | **AOR** | ***95% CI*** | ***p-value*** | ***RE*** |
|  | |  |  |  |  |  |  |  |  |  |  |  |  |  |  |
| ***Fixed component*** | |  |  |  |  |  |  |  |  |  |  |  |  |  |  |
| *Intervention* | |  |  |  |  |  |  |  |  |  |  |  |  |  |  |
|  | No repellent | ref. | - | - | - |  | ref. | - | - | - |  | ref | - | - | - |
|  | Repellent | 0.82 | 0.62,1.09 | 0.180 | - |  | 0.77 | 0.58,1.03 | 0.080 | - |  | 0.75 | 0.55,1.01 | 0.062 | - |
|  | |  |  |  |  |  |  |  |  |  |  |  |  |  |  |
| *Time (month)* | | 0.97 | 0.89,1.07 | 0.582 | - |  | 0.98 | 0.89,1.07 | 0.631 | - |  | 0.98 | 0.89,1.07 | 0.634 | - |
| *Season* | |  |  |  |  |  |  |  |  |  |  |  |  |  |  |
|  | Cool | ref. | - | - | - |  | ref. | - | - | - |  | ref. | - | - | - |
|  | Hot | 1.10 | 0.35,3.44 | 0.871 | - |  | 1.11 | 0.36,3.47 | 0.856 | - |  | 1.12 | 0.36,3.49 | 0.850 | - |
|  | Rainy | 1.17 | 0.45,3.02 | 0.747 | - |  | 1.18 | 0.46,3.03 | 0.738 | - |  | 1.18 | 0.46,3.05 | 0.731 | - |
|  | |  |  |  |  |  |  |  |  |  |  |  |  |  |  |
| ***Random component*** | |  |  |  |  |  |  |  |  |  |  |  |  |  |  |
| $\psi_{1}$^c^ | |  |  |  | 0.52 |  |  |  |  | 0.52 |  |  |  |  | 0.52 |
| $\psi_{2}$ | |  |  |  | 0.13 |  |  |  |  | 0.13 |  |  |  |  | 0.13 |
| $\rho_{11}$^d^ | |  |  |  | 0.03 |  |  |  |  | 0.13 |  |  |  |  | 0.13 |
| $\rho_{12}$^e^ | |  |  |  | 0.16 |  |  |  |  | 0.03 |  |  |  |  | 0.03 |
| $\rho_{2}$^f^ | |  |  |  | 0.13 |  |  |  |  | 0.16 |  |  |  |  | 0.17 |
|  | |  |  |  | -1786.7 |  |  |  |  | *-1786.1* |  |  |  |  | *-1785.9* |
| ***Model fit indices*** | |  |  |  |  |  |  |  |  |  |  |  |  |  |  |
| *AIC* | |  |  |  | 3587.4 |  |  |  |  | 3586.2 |  |  |  |  | 3585.7 |
| *BIC* | |  |  |  | 3639.8 |  |  |  |  | 3638.6 |  |  |  |  | 3638.1 |

Instantaneous and delayed treatment effect comparisons: adjusted odds ratio (AOR), 95% confidence interval (95% CI), probability value (p-value), random-effect variances ($\psi$), conditional intraclass correlation coefficient ($\rho$)^a^ and model log likelihood () from generalised linear mixed modelling (GLMM)^b^

^a^ *ρ* = $\frac{\psi_{k}+ ...+ \psi_{nk}}{\psi_{k}+ ...+ \psi_{nk}+ {\pi^{2}}/3}$ , where $\psi_{k}$ through $\psi_{nk}$ are random-effect (RE) variance estimates pertaining to each of the respective crossed-classified variance components (see table notes ^c-f^) from the crossed random –effect generalised (logit) linear mixed models for a specific ICC estimate.

^b^ Crossed random-effect generalised (logit) linear mixed model with random-effects for temporal-specific (month) and village-specific heterogeneity in infection.

^c^$\psi_{1}$ and $\psi_{2}$ represent variances of the random-effects for month and village respectively.

^d^$\rho_{11}$ represents conditional ICC for participant tests conducted in the same village but different month.

^e^$\rho_{12}$represents conditional ICC for participant tests conducted in the same village and same month.

^f^$\rho_{2}$ represents conditional ICC for participant tests in the same month.
