# Supplementary material for: Evaluation of the effectiveness of topical repellent distributed by village health volunteer networks against Plasmodium spp. infection in Myanmar: A stepped-wedge cluster randomised trial
Source: PLoS Med. 2020 Aug 20;17(8):e1003177. doi: 10.1371/journal.pmed.1003177 (PMC7444540; doi:10.1371/journal.pmed.1003177)
Supplement: S3 Table — (DOCX) [file pmed.1003177.s005.docx]

S3 Table. The instantaneous effect of village repellent distribution on *Plasmodium falciparum* and *Plasmodium vivax* infection using Polymerase Chain Reaction (PCR) detection (n=13,068)

|  | | ***P. falciparum*** | | |  | | ***P. vivax*** | | | | |  | |  |
| --- | --- | --- | --- | --- | --- | --- | --- | --- | --- | --- | --- | --- | --- | --- |
| **Factors** | | **ARRR** | ***95% CI*** | ***p-value*** | |  | | **ARRR** | ***95% CI*** | ***p-value*** |  | | ***RE*** | |
|  | |  |  |  | |  | |  |  |  |  | |  | |
| ***Fixed component*** | |  |  |  | |  | |  |  |  |  | |  | |
|  | |  |  |  | |  | |  |  |  |  | |  | |
| *Intervention* | |  |  |  | |  | |  |  |  |  | |  | |
|  | No repellent | ref. | - | - | |  | | ref. | - | - |  | | - | |
|  | Repellent | 0.67 | 0.47,0.95 | 0.026 | |  | | 1.41 | 0.80,2.47 | 0.233 |  | | - | |
|  | |  |  |  | |  | |  |  |  |  | |  | |
| *Time (month)* | | 1.02 | 0.95,1.11 | 0.523 | |  | | 0.95 | 0.87,1.04 | 0.247 |  | | - | |
|  | |  |  |  | |  | |  |  |  |  | |  | |
| *Season* | |  |  |  | |  | |  |  |  |  | |  | |
|  | Cool | ref. | - | - | |  | | ref. | - | - |  | | - | |
|  | Hot | 0.81 | 0.27,2.40 | 0.700 | |  | | 11.2 | 2.23,56.2 | 0.003 |  | | - | |
|  | Rainy | 0.46 | 0.17,1.21 | 0.115 | |  | | 20.5 | 4.84,86.6 | <0.001 |  | | - | |
|  | |  |  |  | |  | |  |  |  |  | |  | |
| ***Random component*** | |  |  |  | |  | |  |  |  |  | |  | |
|  | |  |  |  | |  | |  |  |  |  | |  | |
| $\psi_{1}$^c^ | |  |  |  | |  | |  |  |  |  | | 0.51 | |
| $\psi_{2}$ | |  |  |  | |  | |  |  |  |  | | 0.14 | |
| $\rho_{11}$^d^ | |  |  |  | |  | |  |  |  |  | | 0.04 | |
| $\rho_{12}$^e^ | |  |  |  | |  | |  |  |  |  | | 0.17 | |
| $\rho_{2}$^f^ | |  |  |  | |  | |  |  |  |  | | 0.13 | |
|  | |  |  |  | |  | |  |  |  |  | | -1660.6 | |
|  | |  |  |  | |  | |  |  |  |  | |  | |
| ***Model fit indices*** | |  |  |  | |  | |  |  |  |  | |  | |
| *AIC* | |  |  |  | |  | |  |  |  |  | | 3345.3 | |
| *BIC* | |  |  |  | |  | |  |  |  |  | | 3434.9 | |

Instantaneous treatment effect comparisons: adjusted relative risk ratio (ARRR), 95% confidence interval (95% CI), probability value (p-value), random-effect variances ($\psi$), conditional intraclass correlation coefficient ($\rho$)^a^ and model log likelihood () from generalised linear mixed modelling (GLMM) using generalised structural equation modelling (GSEM)^b^

^a^ *ρ* = $\frac{\psi_{k}+ ...+ \psi_{nk}}{\psi_{k}+ ...+ \psi_{nk}+ {\pi^{2}}/3}$ , where $\psi_{k}$ through $\psi_{nk}$ are random-effect (RE) variance estimates pertaining to each of the respective crossed-classified variance components (see table notes ^c-f^) from the crossed random–effect generalised (multinomial) linear mixed models for a specific ICC estimate.

^b^ Crossed random-effect generalised (multinomial) linear mixed model with random-effects for temporal-specific (month) and village-specific heterogeneity in infection. No infection was the reference group for the outcome. Random-effects for village and month were constrained to be equal across *Plasmodium* spp. type

^c^$\psi_{1}$ and $\psi_{2}$ represent variances of the random-effects for month and village respectively.

^d^$\rho_{11}$ represents conditional ICC for participant tests conducted in the same village but different month.

^e^$\rho_{12}$represents conditional ICC for participant tests conducted in the same village and same month.

^f^$\rho_{2}$ represents conditional ICC for participant tests in the same month.
