# Supplementary material for: Evaluation of the effectiveness of topical repellent distributed by village health volunteer networks against Plasmodium spp. infection in Myanmar: A stepped-wedge cluster randomised trial
Source: PLoS Med. 2020 Aug 20;17(8):e1003177. doi: 10.1371/journal.pmed.1003177 (PMC7444540; doi:10.1371/journal.pmed.1003177)
Supplement: S4 Table — (DOCX) [file pmed.1003177.s006.docx]

S4 Table. The effect of village repellent distribution on *Plasmodium* spp. infection moderated by participant resident status (PCR) (n=13,157)

| **Factors** | | **AOR** | ***95% CI*** | ***p-value*** | ***RE*** |
| --- | --- | --- | --- | --- | --- |
|  | |  |  |  |  |
| ***Fixed component*** | |  |  |  |  |
|  | |  |  |  |  |
| *Intervention* | |  |  |  |  |
|  | No repellent | ref. | - | - | - |
|  | Repellent | 0.65 | 0.44,.96 | 0.032 |  |
|  |  |  |  |  |  |
| *Resident status* | |  |  |  |  |
|  | Resident | ref. | - | - | - |
|  | Migrant | 0.91 | 0.61,1.37 | 0.661 |  |
|  | Forest Dweller | 0.91 | 0.68,1.20 | 0.501 |  |
|  | |  |  |  |  |
| *Intervention by resident status^c^* | |  |  |  |  |
|  | Migrant | 1.74 | 0.89,3.40 | 0.105 | - |
|  | Forest Dweller | 1.38 | 0.88,2.16 | 0.159 | - |
|  |  |  |  |  |  |
| *Time (month)* | | 0.98 | 0.89,1.07 | 0.612 | - |
|  | |  |  |  |  |
| *Season* | |  |  |  |  |
|  | Cool | ref. | - | - | - |
|  | Hot | 1.10 | 0.35,3.46 | 0.866 | - |
|  | Rainy | 1.17 | 0.45,3.02 | 0.749 | - |
|  | |  |  |  |  |
| ***Random component*** | |  |  |  |  |
|  | |  |  |  |  |
| $\psi_{1}$^d^ | |  |  |  | 0.52 |
| $\psi_{2}$ | |  |  |  | 0.13 |
| $\rho_{11}$^e^ | |  |  |  | 0.03 |
| $\rho_{12}$^f^ | |  |  |  | 0.16 |
| $\rho_{2}$^g^ | |  |  |  | 0.13 |
|  | |  |  |  | *-1784.8* |
|  | |  |  |  |  |

Instantaneous treatment effect of differing levels of average usage: adjusted odds ratio (AOR), 95% confidence interval (95% CI), probability value (p-value), random-effect variances ($\psi$), conditional intraclass correlation coefficient ($\rho$)^a^ and model log likelihood () from generalised linear mixed modelling (GLMM)^b^

^a^ *ρ* = $\frac{\psi_{k}+ ...+ \psi_{nk}}{\psi_{k}+ ...+ \psi_{nk}+ {\pi^{2}}/3}$ , where $\psi_{k}$ through $\psi_{nk}$ are random-effect (RE) variance estimates pertaining to each of the respective crossed-classified variance components (see table notes ^c-f^) from the crossed random–effect generalised (logit) linear mixed models for a specific ICC estimate.

^b^ Crossed random-effect generalised (logit) linear mixed model (logit link function and binomial distribution) with random-effects for temporal-specific (month) and village-specific heterogeneity in infection.

^c^ Intervention by resident status interaction term – represents the multiplicative effect on the intervention of having either migrant or forest dweller resident status relative to residents.

^d^$\psi_{1}$and $\psi_{2}$ represent variances of the random-effects for month and village respectively.

^e^$\rho_{11}$ represents conditional ICC for participant tests conducted in the same village but different month in a control period.

^f^$\rho_{12}$represents conditional ICC for participant tests conducted in the same village and same month in a control period.

^g^$\rho_{2}$ represents conditional ICC for participant tests in the same month.
