# Supplementary material for: Evaluation of the effectiveness of topical repellent distributed by village health volunteer networks against Plasmodium spp. infection in Myanmar: A stepped-wedge cluster randomised trial
Source: PLoS Med. 2020 Aug 20;17(8):e1003177. doi: 10.1371/journal.pmed.1003177 (PMC7444540; doi:10.1371/journal.pmed.1003177)
Supplement: S5 Table — (DOCX) [file pmed.1003177.s007.docx]

S5 Table. The effect of village repellent distribution on *Plasmodium* *falciparum* and *Plasmodium vivax* infection moderated by participant resident status (PCR) (n=13,068)

|  | | ***P. falciparum*** | | |  | | ***P. vivax*** | | | | |  | |  |
| --- | --- | --- | --- | --- | --- | --- | --- | --- | --- | --- | --- | --- | --- | --- |
| **Factors** | | **ARRR** | ***95% CI*** | ***p-value*** | |  | | **ARRR** | ***95% CI*** | ***p-value*** |  | | ***RE*** | |
|  | |  |  |  | |  | |  |  |  |  | |  | |
| ***Fixed component*** | |  |  |  | |  | |  |  |  |  | |  | |
|  | |  |  |  | |  | |  |  |  |  | |  | |
| *Intervention* | |  |  |  | |  | |  |  |  |  | |  | |
|  | No repellent | ref. | - | - | |  | | ref. | - | - |  | | - | |
|  | Repellent | 0.58 | 0.38,0.88 | 0.011 | |  | | 1.17 | 0.58,2.36 | 0.653 |  | | - | |
|  | |  |  |  | |  | |  |  |  |  | |  | |
| *Resident status* | |  |  |  | |  | |  |  |  |  | |  | |
|  | Resident | ref | - | - | |  | | ref | - | - |  | | - | |
|  | Migrant | 1.10 | 0.62,1.93 | 0.747 | |  | | 0.82 | 0.40,1.67 | 0.586 |  | | - | |
|  | Forest Dweller | 0.91 | 0.59,1.39 | 0.649 | |  | | 0.76 | 0.46,1.24 | 0.272 |  | | - | |
|  | |  |  |  | |  | |  |  |  |  | |  | |
| *Intervention by resident status^c^* | |  |  |  | |  | |  |  |  |  | |  | |
|  | Migrant | 1.34 | 0.56,3.19 | 0.513 | |  | | 1.74 | 0.53,5.79 | 0.364 |  | | - | |
|  | Forest Dweller | 1.29 | 0.74,2.24 | 0.374 | |  | | 1.27 | 0.58,2.80 | 0.551 |  | | - | |
|  | |  |  |  | |  | |  |  |  |  | |  | |
| *Time (month)* | | 1.03 | 0.94,1.12 | 0.560 | |  | | 0.95 | 0.88,1.03 | 0.200 |  | | - | |
|  | |  |  |  | |  | |  |  |  |  | |  | |
| *Season* | |  |  |  | |  | |  |  |  |  | |  | |
|  | Cool | ref. | - | - | |  | | ref. | - | - |  | | - | |
|  | Hot | 0.81 | 0.26,2.52 | 0.719 | |  | | 11.4 | 2.71,47.5 | 0.001 |  | | - | |
|  | Rainy | 0.46 | 0.18,1.14 | 0.093 | |  | | 20.8 | 6.33,68.2 | <0.001 |  | | - | |
|  | |  |  |  | |  | |  |  |  |  | |  | |
| ***Random component*** | |  |  |  | |  | |  |  |  |  | |  | |
|  | |  |  |  | |  | |  |  |  |  | |  | |
| $\psi_{1}$^d^ | |  |  |  | |  | |  |  |  |  | | 0.51 | |
| $\psi_{2}$ | |  |  |  | |  | |  |  |  |  | | 0.13 | |
| $\rho_{11}$^e^ | |  |  |  | |  | |  |  |  |  | | 0.03 | |
| $\rho_{12}$^f^ | |  |  |  | |  | |  |  |  |  | | 0.16 | |
| $\rho_{2}$^g^ | |  |  |  | |  | |  |  |  |  | | 0.13 | |
|  | |  |  |  | |  | |  |  |  |  | | -1658.9 | |
|  | |  |  |  | |  | |  |  |  |  | |  | |
| ***Model fit indices*** | |  |  |  | |  | |  |  |  |  | |  | |
| *AIC* | |  |  |  | |  | |  |  |  |  | | 3357.8 | |
| *BIC* | |  |  |  | |  | |  |  |  |  | | 3507.4 | |

Instantaneous treatment effect comparisons moderated by resident status: adjusted relative risk ratio (ARRR), 95% confidence interval (95% CI), probability value (p-value), random-effect variances ($\psi$), conditional intraclass correlation coefficient ($\rho$)^a^ and model log likelihood () from generalised linear mixed modelling (GLMM) using generalised structural equation modelling (GSEM)^b^

^a^ *ρ* = $\frac{\psi_{k}+ ...+ \psi_{nk}}{\psi_{k}+ ...+ \psi_{nk}+ {\pi^{2}}/3}$ , where $\psi_{k}$ through $\psi_{nk}$ are random-effect (RE) variance estimates pertaining to each of the respective crossed-classified variance components (see table notes ^c-f^) from the crossed random–effect generalised (multinomial) linear mixed models for a specific ICC estimate.

^c^ Intervention by resident status interaction term – represents the multiplicative effect on the intervention of having either migrant or forest dweller resident status relative to residents.

^d^$\psi_{1}$ and $\psi_{2}$ represent variances of the random-effects for month and village respectively.

^e^$\rho_{11}$ represents conditional ICC for participant tests conducted in the same village but different month.

^f^$\rho_{12}$represents conditional ICC for participant tests conducted in the same village and same month.

^g^$\rho_{2}$ represents conditional ICC for participant tests in the same month.
