# Supplementary material for: Evaluation of the effectiveness of topical repellent distributed by village health volunteer networks against Plasmodium spp. infection in Myanmar: A stepped-wedge cluster randomised trial
Source: PLoS Med. 2020 Aug 20;17(8):e1003177. doi: 10.1371/journal.pmed.1003177 (PMC7444540; doi:10.1371/journal.pmed.1003177)
Supplement: S6 Table — (DOCX) [file pmed.1003177.s008.docx]

S6 Table. The delayed effect of village repellent distribution on *Plasmodium falciparum* and *Plasmodium vivax* infection using Polymerase Chain Reaction (PCR) detection (n=13,068)

|  | | ***1-month delay*** | | | | | | | | |  | ***2-month delay*** | | | | | | | | |
| --- | --- | --- | --- | --- | --- | --- | --- | --- | --- | --- | --- | --- | --- | --- | --- | --- | --- | --- | --- | --- |
|  | | ***P. falciparum*** | | |  | ***P. vivax*** | | |  | ***RE*** |  | ***P. falciparum*** | | |  | ***P. vivax*** | | |  | ***RE*** |
| **Factors** | | **ARRR** | ***95%CI*** | ***p-value*** |  | **ARRR** | ***95%CI*** | ***p-value*** |  |  |  | **ARRR** | ***95%CI*** | ***p-value*** |  | **ARRR** | ***95%CI*** | ***p-value*** |  |  |
|  | |  |  |  |  |  |  |  |  |  |  |  |  |  |  |  |  |  |  |  |
| ***Fixed***  ***component*** | |  |  |  |  |  |  |  |  |  |  |  |  |  |  |  |  |  |  |  |
|  | |  |  |  |  |  |  |  |  |  |  |  |  |  |  |  |  |  |  |  |
| *Intervention* | |  |  |  |  |  |  |  |  |  |  |  |  |  |  |  |  |  |  |  |
|  | No repellent | ref. | - | - |  | ref. | - | - |  | **-** |  | ref. | - | - |  | ref. | - | - |  | **-** |
|  | Repellent | 0.77 | 0.53,1.11 | 0.163 |  | 0.97 | 0.57,1.65 | 0.910 |  | **-** |  | 0.81 | 0.55,1.17 | 0.252 |  | 0.77 | 0.40,1.50 | 0.444 |  | **-** |
|  | |  |  |  |  |  |  |  |  |  |  |  |  |  |  |  |  |  |  |  |
| *Time (month)* | | 1.01 | 0.92,1.12 | 0.785 |  | 0.97 | 0.89,1.06 | 0.511 |  | **-** |  | 1.01 | 0.92,1.11 | 0.846 |  | 0.99 | 0.88,1.10 | 0.796 |  | **-** |
|  | |  |  |  |  |  |  |  |  |  |  |  |  |  |  |  |  |  |  |  |
| *Season* | |  |  |  |  |  |  |  |  |  |  |  |  |  |  |  |  |  |  |  |
|  | Cool | ref. | - | - |  | ref. | - | - |  | **-** |  | ref. | - | - |  | ref. | - | - |  | **-** |
|  | Hot | 0.81 | 0.29,2.26 | 0.688 |  | 11.0 | 3.44,35.5 | <0.001 |  | - |  | 0.81 | 0.24,2.70 | 0.732 |  | 11.2 | 2.1,60.8 | <0.001 |  | - |
|  | Rainy | 0.46 | 0.22,0.96 | 0.039 |  | 20.2 | 8.56,47.5 | <0.001 |  | - |  | 0.46 | 0.17,1.22 | 0.120 |  | 20.3 | 4.74,86.9 | <0.001 |  | - |
|  | |  |  |  |  |  |  |  |  |  |  |  |  |  |  |  |  |  |  |  |
| ***Random***  ***component*** | |  |  |  |  |  |  |  |  |  |  |  |  |  |  |  |  |  |  |  |
|  | |  |  |  |  |  |  |  |  |  |  |  |  |  |  |  |  |  |  |  |
| $\psi_{1}$^c^ | |  |  |  |  |  |  |  |  | 0.51 |  |  |  |  |  |  |  |  |  | 0.51 |
| $\psi_{2}$ | |  |  |  |  |  |  |  |  | 0.14 |  |  |  |  |  |  |  |  |  | 0.14 |
| $\rho_{11}$^d^ | |  |  |  |  |  |  |  |  | 0.03 |  |  |  |  |  |  |  |  |  | 0.03 |
| $\rho_{12}$^e^ | |  |  |  |  |  |  |  |  | 0.13 |  |  |  |  |  |  |  |  |  | 0.13 |
| $\rho_{2}$^f^ | |  |  |  |  |  |  |  |  | 0.16 |  |  |  |  |  |  |  |  |  | 0.16 |
|  | |  |  |  |  |  |  |  |  | -1663.0 |  |  |  |  |  |  |  |  |  | -1663.1 |
|  | |  |  |  |  |  |  |  |  |  |  |  |  |  |  |  |  |  |  |  |
| ***Model fit indices*** | |  |  |  |  |  |  |  |  |  |  |  |  |  |  |  |  |  |  |  |
| *AIC* | |  |  |  |  |  |  |  |  | 3350.0 |  |  |  |  |  |  |  |  |  | 3350.3 |
| *BIC* | |  |  |  |  |  |  |  |  | 3439.8 |  |  |  |  |  |  |  |  |  | 3440.0 |

Delayed treatment effect comparisons: adjusted relative risk ratio (ARRR), 95% confidence interval (95% CI), probability value (p-value), random-effect variances ($\psi$), conditional intraclass correlation coefficient ($\rho$)^a^ and model log likelihood () from generalised linear mixed modelling (GLMM) using generalised structural equation modelling (GSEM)^b^

^a^ *ρ* = $\frac{\psi_{k}+ ...+ \psi_{nk}}{\psi_{k}+ ...+ \psi_{nk}+ {\pi^{2}}/3}$ , where $\psi_{k}$ through $\psi_{nk}$ are random-effect (RE) variance estimates pertaining to each of the respective crossed-classified variance components (see table notes ^c-f^) from the crossed random–effect generalised (multinomial) linear mixed models for a specific ICC estimate.

^b^ Crossed random-effect generalised (multinomial) linear mixed model with random-effects for temporal-specific (month) and village-specific heterogeneity in infection. No infection was the reference group for the outcome. Random-effects for village and month were constrained to be equal across *Plasmodium* spp. type

^c^$\psi_{1}$ and $\psi_{2}$ represent variances of the random-effects for month and village respectively.

^d^$\rho_{11}$ represents conditional ICC for participant tests conducted in the same village but different month.

^e^$\rho_{12}$represents conditional ICC for participant tests conducted in the same village and same month.

^f^$\rho_{2}$ represents conditional ICC for participant tests in the same month.
