# Supplementary material for: Evaluation of the effectiveness of topical repellent distributed by village health volunteer networks against Plasmodium spp. infection in Myanmar: A stepped-wedge cluster randomised trial
Source: PLoS Med. 2020 Aug 20;17(8):e1003177. doi: 10.1371/journal.pmed.1003177 (PMC7444540; doi:10.1371/journal.pmed.1003177)
Supplement: S7 Table — (DOCX) [file pmed.1003177.s009.docx]

S7 Table. Analysis of adherence to intervention (per protocol analysis): The instantaneous effect of village repellent distribution with differing levels of average usage on Plasmodium species infection (RDT): (n=28,408)

| **Factors** | | **AOR** | ***95% CI*** | ***p-value*** | ***RE*** |
| --- | --- | --- | --- | --- | --- |
|  | |  |  |  |  |
| ***Fixed component*** | |  |  |  |  |
|  | |  |  |  |  |
| *Intervention* | |  |  |  |  |
|  | No repellent | ref. | - | - | - |
|  | Repellent – monthly | 1.54 | 0.14,16.7 | 0.722 |  |
|  | Repellent – weekly | 0.33 | 0.01,22.2 | 0.604 | - |
|  | Repellent – daily | 0.05 | 0.0002,10.3 | 0.272 | - |
|  | |  |  |  |  |
| *Time (month)* | | 0.87 | 0.77,0.99 | 0.029 | - |
|  | |  |  |  |  |
| *Season* | |  |  |  |  |
|  | Cool | ref. | - | - | - |
|  | Hot | 3.57 | 0.64,19.8 | 0.072 | - |
|  | Rainy | 4.67 | 1.02,21.4 | 0.048 | - |
|  | |  |  |  |  |
| ***Random component*** | |  |  |  |  |
|  | |  |  |  |  |
| $\psi_{1}$^c^ | |  |  |  | 0.042 |
| $\psi_{2}$ | |  |  |  | 2.78 |
| $\psi_{3}$ | |  |  |  | 1.77 |
| $\rho_{01}$^d^ | |  |  |  | 0.45 |
| $\rho_{02}$^e^ | |  |  |  | 0.46 |
| $\rho_{11}$^f^ | |  |  |  | 0.58 |
| $\rho_{12}$^g^ | |  |  |  | 0.58 |
| $\rho_{2}$^h^ | |  |  |  | 0.007 |
|  | |  |  |  | *-311.86* |
|  | |  |  |  |  |

Instantaneous treatment effect differing levels of average usage: adjusted odds ratio (AOR), 95% confidence interval (95% CI), probability value (p-value), random-effect variances ($\psi$), conditional intraclass correlation coefficient ($\rho$)^a^ and model log likelihood () from generalised linear mixed modelling (GLMM)^b^

^a^ *ρ* = $\frac{\psi_{k}+ ...+ \psi_{nk}}{\psi_{k}+ ...+ \psi_{nk}+ {\pi^{2}}/3}$ , where $\psi_{k}$ through $\psi_{nk}$ are random-effect (RE) variance estimates pertaining to each of the respective crossed-classified variance components (see table notes ^c-h^) from the crossed random–effect generalised (logit) linear mixed models for a specific ICC estimate.

^b^ Crossed random-effect generalised (logit) linear mixed model (logit link function and binomial distribution) with random-effects for temporal-specific (month), village-specific heterogeneity in infection and village-specific heterogeneity in effect of repellent distribution. Likelihood ratio tests did not support a less-constrained model whereby a covariance term for village-specific probability of Plasmodium infection and heterogeneity in intervention effect was estimated.

^c^$\psi_{1}$, $\psi_{2}$ and $\psi_{3}$ represent variances of the random-effects for month, village and intervention respectively.

^d^$\rho_{01}$ represents conditional ICC for participant tests conducted in the same village but different month in a control period.

^e^$\rho_{02}$represents conditional ICC for participant tests conducted in the same village and same month in a control period.

^f^$\rho_{11}$ represents conditional ICC for participant tests conducted in the same village but different month in an intervention period.

^g^$\rho_{12}$ represents conditional ICC for participant tests conducted in the same village and same month in an intervention period.

^h^$\rho_{2}$ represents conditional ICC for participant tests in the same month.
