# Supplementary material for: Evaluation of the effectiveness of topical repellent distributed by village health volunteer networks against Plasmodium spp. infection in Myanmar: A stepped-wedge cluster randomised trial
Source: PLoS Med. 2020 Aug 20;17(8):e1003177. doi: 10.1371/journal.pmed.1003177 (PMC7444540; doi:10.1371/journal.pmed.1003177)
Supplement: S8 Table — (DOCX) [file pmed.1003177.s010.docx]

S8 Table. Analysis of adherence to intervention (per protocol analysis): The instantaneous effect of village mosquito repellent distribution with differing levels of average usage on Plasmodium spp. infection (PCR) (n=11,833)

| **Factors** | | **AOR** | ***95% CI*** | ***p-value*** | ***RE*** |
| --- | --- | --- | --- | --- | --- |
|  | |  |  |  |  |
| ***Fixed component*** | |  |  |  |  |
|  | |  |  |  |  |
| *Intervention* | |  |  |  |  |
|  | No repellent | ref. | - | - | - |
|  | Repellent – monthly | 1.22 | 0.80,1.86 | 0.365 |  |
|  | Repellent – weekly | 0.68 | 0.41,1.15 | 0.152 | - |
|  | Repellent – daily | 0.86 | 0.60,1.21 | 0.379 | - |
|  | |  |  |  |  |
| *Time (month)* | | 0.97 | 0.88,1.07 | 0.587 | - |
|  | |  |  |  |  |
| *Season* | |  |  |  |  |
|  | Cool | ref. | - | - | - |
|  | Hot | 1.13 | 0.35,3.65 | 0.839 | - |
|  | Rainy | 1.20 | 0.45,3.20 | 0.709 | - |
|  | |  |  |  |  |
| ***Random component*** | |  |  |  |  |
|  | |  |  |  |  |
| $\psi_{1}$^c^ | |  |  |  | 0.54 |
| $\psi_{2}$ | |  |  |  | 0.13 |
| $\rho_{11}$^d^ | |  |  |  | 0.03 |
| $\rho_{12}$^e^ | |  |  |  | 0.17 |
| $\rho_{2}$^f^ | |  |  |  | 0.14 |
|  | |  |  |  | *-1586.8* |
|  | |  |  |  |  |

Instantaneous treatment effect differing levels of average usage: adjusted odds ratio (AOR), 95% confidence interval (95% CI), probability value (p-value), random-effect variances ($\psi$), conditional intraclass correlation coefficient ($\rho$)^a^ and model log likelihood () from generalised linear mixed modelling (GLMM)^b^

^a^ *ρ* = $\frac{\psi_{k}+ ...+ \psi_{nk}}{\psi_{k}+ ...+ \psi_{nk}+ {\pi^{2}}/3}$ , where $\psi_{k}$ through $\psi_{nk}$ are random-effect (RE) variance estimates pertaining to each of the respective crossed-classified variance components (see table notes ^c-f^) from the crossed random–effect generalised (logit) linear mixed models for a specific ICC estimate.

^b^ Crossed random-effect generalised (logit) linear mixed model (logit link function and binomial distribution) with random-effects for temporal-specific (month) and village-specific heterogeneity in infection.

^c^$\psi_{1}$and $\psi_{2}$ represent variances of the random-effects for month and village respectively.

^d^$\rho_{11}$ represents conditional ICC for participant tests conducted in the same village but different month in a control period.

^e^$\rho_{12}$represents conditional ICC for participant tests conducted in the same village and same month in a control period.

^f^$\rho_{2}$ represents conditional ICC for participant tests in the same month.
