# Supplementary material for: Evaluation of the effectiveness of topical repellent distributed by village health volunteer networks against Plasmodium spp. infection in Myanmar: A stepped-wedge cluster randomised trial
Source: PLoS Med. 2020 Aug 20;17(8):e1003177. doi: 10.1371/journal.pmed.1003177 (PMC7444540; doi:10.1371/journal.pmed.1003177)
Supplement: S1 Text — (DOCX) [file pmed.1003177.s011.docx]

**S1 Text. Guidelines for Repellent Usage**

What is a mosquito repellent?

- A mosquito repellent is a substance applied to skin, clothing, or other surfaces which discourages mosquitoes from landing or climbing on that surface.

How do mosquito repellents work?

- Mosquito repellents do not kill mosquitoes. If you apply them on your body, they prevent mosquitoes from biting you for a period of time. How long repellents stay effective depends on the nature of the chemicals present and how humid the weather is.
- Repellents made from chemicals and plants are available in the form of creams, lotions, sprays, wipes, roll-on sticks, foams, liquid vaporizers, sprays, coils and mats.

What is this repellent cream?

- Clinically tested and certified, repellent cream offers the effective defense against mosquitoes for as long as 8 hours. Repellent cream is equally effective against outdoors and daytime mosquitoes too. By using this cream, it can prevent major mosquito borne diseases like malaria, dengue, chikungunya, and Japanese B encephalitis.

How to apply?

- To get full protection from mosquitoes, apply repellent to your hands first, and then rub with the hands over exposed parts of the skin. Avoid application on or near eyes, mouth, and open wounds.
- Volume to be applied should be judged to equally distribute the cream over the skin with single layer.
- Apply the repellent in evening such as at around 5 pm daily. The effect of the repellent typically lasts for eight hours. Therefore, repellent needs to be reapplied after eight hours.
- It is safe to use mosquito repellent cream to babies six months old and above. For babies younger than six months, repellent must not be used. Bed net should be used for them.
- Keep mosquito repellent at a separate location and out of the reach of children. It should not be kept together with other medicines and cosmetics.

What are the risks of applying repellent?

- The risks include allergy and ingestion.

Whom to inform?

- Inform village health volunteer (VHV) immediately when allergy or ingestion occurs
- Inform VHV before you run out of repellent and bring the empty tubes for replenishment
- Inform VHV in case of lost or damage of repellent
