# Supplementary material for: Evaluation of the effectiveness of topical repellent distributed by village health volunteer networks against Plasmodium spp. infection in Myanmar: A stepped-wedge cluster randomised trial
Source: PLoS Med. 2020 Aug 20;17(8):e1003177. doi: 10.1371/journal.pmed.1003177 (PMC7444540; doi:10.1371/journal.pmed.1003177)
Supplement: S2 Text — (DOCX) [file pmed.1003177.s012.docx]

S2 Text. Molecular determination of *Plasmodium* spp. infections

qPCR detection of *Plasmodium* spp. infections

DNA was extracted from DBS pools consisting of a single 3mm punch from 10 separate participant samples using the FavorPrep 96-well genomic DNA extraction kit by vacuum manifold according to manufacturer’s instructions. DNA was eluted in 50µL elution buffer. Pooled DNA was then tested for generic *Plasmodium* spp. infection in simplex qPCR (summarised in the tables below).

qPCR detection of *P. falciparum* and *P. vivax* infections

DNA from samples included in a pool that was positive for *Plasmodium* spp. infection was then extracted from 2 x 3mm DBS punches and utilised in duplex qPCR for *P. falciparum* and *P. vivax* (Summarised in Tables below) according to previously published methods [1]. Standard curve for *P. falciparum and P. vivax* was made from a 10-fold serial dilution of the control plasmids ranging from 10^5^ copies/μL to 5 copies/μL in duplicate. Samples yielding threshold cycle values equal or higher than 40 were considered *Plasmodium* species negative. All assays were run in 96-well plate format on a Stratagene x3005p lightcycler.

qPCR primers and probes

| **Species** | **Fluorescent label** | **Primer/probe** | **Sequence 5'-3'** |
| --- | --- | --- | --- |
| *Plasmodium* spp. | FAM-BHQ | Forward | CTTCCTTAGATGTGGTAGCTATTTCTCA |
| *“* | “ | Reverse | ACATGGCTATGACGGGTAAC |
| *“* | “ | Probe | FAMAATTAGAGTTCGATTC |
| *P. falciparum* | FAM-BHQ | Forward | TATTGCTTTTGAGAGGTTTTGTTACTTTG |
| “ | “ | Reverse | ACCTCTGACATCTGAATACGAATGC |
| “ | “ | Probe | ACGGGTAGTCATGATTGAGTT |
| *P. vivax* | HEX-BHQ | Forward | GCTTTGTAATTGGAATGATGGGAAT |
| “ | “ | Reverse | ATGCGCACAAAGTCGATACGAAG |
| “ | “ | Probe | AGCAACGCTTCTAGCTTA |

qPCR reagents and cycling conditions

| ***Plasmodium* spp.** | | | |  | ***P. falciparum* and *P. vivax*** | | | |
| --- | --- | --- | --- | --- | --- | --- | --- | --- |
| **Reagent** | **µL/reaction** | **Cycling parameters** | **Cycles** |  | **Reagent** | **µL/reaction** | **Cycling parameters** | **Cycles** |
| Taqman master mix | 10 | 95°C 2 minutes |  |  | Taqman master mix | 6.5 | 95°C 15 minutes |  |
| Forward primer | 0.8 |  |  |  | Forward primer | 0.2 |  |  |
| Reverse primer | 0.8 | 95°C 10 seconds | 40 |  | Reverse primer | 0.2 | 95°C 15 seconds | 45 |
| Probe | 0.2 | 60°C 50 seconds |  |  | Probe | 0.45 | 60°C 1 minute |  |
| Nuclease free water | 3.2 |  |  |  | Nuclease free water | 0.8 | 40°C 1 minute |  |
| DNA | 5 |  |  |  | DNA | 4 |  |  |

**References**

1. Rosanas-Urgell A, Mueller D, Betuela I, Barnadas C, Iga J, Zimmerman PA, et al. Comparison of diagnostic methods for the detection and quantification of the four sympatric *Plasmodium* species in field samples from Papua New Guinea. Malar J. 2010;9(1):361. doi: 10.1186/1475-2875-9-361.
