# Supplementary material for: Evaluation of the effectiveness of topical repellent distributed by village health volunteer networks against Plasmodium spp. infection in Myanmar: A stepped-wedge cluster randomised trial
Source: PLoS Med. 2020 Aug 20;17(8):e1003177. doi: 10.1371/journal.pmed.1003177 (PMC7444540; doi:10.1371/journal.pmed.1003177)
Supplement: S3 Text — (DOCX) [file pmed.1003177.s013.docx]

S3 Text. Statistical notation for the generalised linear mixed model used to estimate the effect of repellent distribution on *Plasmodium* spp. infection.

The model can be formally written as:

${logit\{\text{Pr(}y}_{ij}=1)\left| x_{ij}, \zeta_{1j,}\zeta_{2i,} \zeta_{3j}{Intervention}_{i-tj} \right\}= \beta_{1}+ \beta_{2}{{Intervention}_{i-tj}+{\beta_{3}Time}_{ij}+{\beta_{4}Season}_{1ij}+ {\beta_{5}Season}_{2ij}+\zeta}_{1j}+ {\zeta_{2i}+ \zeta}_{3j}{Intervention}_{i-tj}$, (1)

where

$\zeta_{1j}$ ~ *N*(0,$\psi_{1}$), $\zeta_{2i}$ ~ *N*(0,$\psi_{2}$) and $\zeta_{3j}{Intervention}_{ij}$ ~ *N*(0,$\psi_{3}$) , (2)

Where $x_{ij}$ is the vector of model time-varying covariates, $\beta_{1}$ is the model constant and represents the probability of infection at baseline and during ‘cool’ season, $\beta_{2}$ the time-varying fixed effect for repellent distribution for village *j* at occasion *i-t* (where *t* is time (months) since the introduction of repellent), $\beta_{3}$ the linear effect of time, $\beta_{4}$and $\beta_{5}$dummy indicators for the effect of malaria season, $\zeta_{1j}$the random-effect (i.e. intercept) for between-village heterogeneity in baseline probability of malaria, $\zeta_{3j}$ the random-effect (i.e. coefficient) for between-village heterogeneity in the effect of repellent distribution and $\zeta_{2i,}$ the random-effect (i.e. intercept) for the temporal (i.e. between-month) variability in probability of malaria.
