# Supplementary material for: Evaluation of the effectiveness of topical repellent distributed by village health volunteer networks against Plasmodium spp. infection in Myanmar: A stepped-wedge cluster randomised trial
Source: PLoS Med. 2020 Aug 20;17(8):e1003177. doi: 10.1371/journal.pmed.1003177 (PMC7444540; doi:10.1371/journal.pmed.1003177)
Supplement: S4 Text — (DOCX) [file pmed.1003177.s014.docx]

S4 Text. Statistical analysis plan taken from published study protocol

**Statistical analysis**

Quantitative data collected in the field will be entered into a database and subsequently imported into Stata for necessary data management and analysis. Difference in risk of the primary outcome *P. falciparum* and *P. vivax* incidence will be estimated across intervention and control states using a generalised linear maximum likelihood mixed modelling (GLMM, a.k.a. multi-level modelling) analytical approach, with village clusters treated as a random effect and study group, time and seasonality estimated as independent fixed factors. Temporal and spatial trends of *P. falciparum* and *P. vivax* incidence will also be explored including analysis of the effectiveness of the intervention across the study period. Sandwich estimator variance estimation will be used to adjust for potential lack of independence in observations within clusters over the study period. Generalised linear mixed modelling will also be extended to facilitate modelling of both village and temporal (cross-sectional month) dependence underpinning *P. falciparum* and *P. vivax* infection incidence and the extent to which between-village heterogeneity is present in the effect of repellent distribution on incidence of these infections. Analyses of secondary outcomes will also involve multi-level modelling (i.e. linear mixed modelling (LLM) and GLMM). Analyses of the implementation will also be performed such as exploring rates of repellent uptake, reported side effects and adverse events (e.g. allergic reactions).
