# Supplementary material for: Evaluation of the effectiveness of topical repellent distributed by village health volunteer networks against Plasmodium spp. infection in Myanmar: A stepped-wedge cluster randomised trial
Source: PLoS Med. 2020 Aug 20;17(8):e1003177. doi: 10.1371/journal.pmed.1003177 (PMC7444540; doi:10.1371/journal.pmed.1003177)
Supplement: S5 Text — (DOCX) [file pmed.1003177.s015.docx]

S5 Text. Repellent costs.

Itemised cost (2015 US$) to provide 19,808 person-years of topical insect repellent

| **Item** | **US$** |
| --- | --- |
| Tubes of insect repellent | 28,139 |
| Staff costs | 45,172 |
| Rent | 1,257 |
| Distribution | 1,524 |
| Meetings | 47 |
| Total | 76,138 |

We assessed the costs associated with adding repellent distribution to malaria activities that were already being implemented at the villages. Any costs related to the study design were excluded. In addition to the cost of the tubes of repellent, the costs of distribution, staff, rent for facilities, and meetings were included. Since the total cost of these activities was attributed to a range of activities, the proportion needed for repellent distribution was estimated for each. All costs are reported in 2015 United States Dollars (US$).

For the effectiveness measures, the number of people-years of coverage was calculated using the village population and the number of months each village was receiving the repellent. For villages where the population was not known, the mean number of people in a village was used; taken from census data (based on villages where a census has been undertaken). This resulted in 19,808 person-years of repellent coverage

The cost-effectiveness was determined using a hypothetical cohort of 10,000 individuals. The total cost of adding repellent distribution was estimated using the cost per person-year in the study. To determine the number of sub-microscopic cases of any species averted by repellent distribution, predicted probabilities (including random-effects) under both control and intervention states were estimated from the *Plasmodium* spp. infection GLMM, with the effect of repellent distribution constrained to the magnitude of effect (33% reduction) observed in *P. falciparum* modelling. These probabilities were multiplied by the hypothetical cohort size to estimate cases under both control and intervention conditions; then used to estimate an incremental cost-effectiveness ratio (ICER, see formula below). Since we observed village-specific heterogeneity in malaria prevalence, a sensitivity analysis was undertaken to explore the impact of varying prevalence on the ICER. In order to assess impact in the context of the study population, we estimated ICERs for the lower and upper limits of the 95% reference range for village-specific malaria probabilities. Village-specific malaria probabilities were derived from the linear combination of empirical Bayes (village-specific) random intercept predictions and the average probability of malaria infection (fixed intercept) produced from the effect constrained *Plasmodium* spp GLMM.

The formula for the incremental cost-effectiveness ratio (ICER) is:

$$ICER = \frac{{Cost}_{r}- {Cost}_{u}}{{Cases}_{u}-{Cases}_{r}}$$

Where *r* designates the repellent intervention and *u* designates usual care
