# Supplementary material for: Evaluation of the effectiveness of topical repellent distributed by village health volunteer networks against Plasmodium spp. infection in Myanmar: A stepped-wedge cluster randomised trial
Source: PLoS Med. 2020 Aug 20;17(8):e1003177. doi: 10.1371/journal.pmed.1003177 (PMC7444540; doi:10.1371/journal.pmed.1003177)
Supplement: S6 Text — (DOCX) [file pmed.1003177.s016.docx]

S6 Text. Results of inferential tests assessing heterogeneity in the effect of repellent distribution using generalised linear mixed modelling.

As specified in notation 2) of S3 Text, likelihood ratio tests indicated that a less-constrained model relaxing the constraint of a common effect of repellent distribution on *Plasmodium* spp. infection was appropriate for RDT data (Likelihood Ratio [LR] χ^2^ (1) = 4.66, p=0.031), although there was no empirical evidence for (unstructured) covariance (or in other words an association) between the observed village-specific heterogeneity in repellent effect and village-specific baseline probability of *Plasmodium* spp. infection (LR χ^2^ (1) = 1.78, p=0.182). Unlike the results from statistical modelling of the effect of the intervention on *Plasmodium* spp. infection using RDT, there was no evidence for intervention effect heterogeneity across villages (i.e. random coefficient model) with respect to *Plasmodium* spp. infection by PCR (LR χ^2^(1) = 0.15, p=0.699).
